# Silactins and Structural Diversity of Biosilica in Sponges

**DOI:** 10.3390/biomimetics9070393

**Published:** 2024-06-27

**Authors:** Hermann Ehrlich, Alona Voronkina, Konstantin Tabachniсk, Anita Kubiak, Alexander Ereskovsky, Teofil Jesionowski

**Affiliations:** 1 Center of Advanced Technology, Adam Mickiewicz University, Uniwersytetu Poznańskiego 10, 61-614 Poznan, Poland; anita.kubiak@amu.edu.pl; 2 Faculty of Chemical Technology, Institute of Chemical Technology and Engineering, Poznan University of Technology, Berdychowo 4, 60-965 Poznan, Poland; 3 Pharmacy Department, National Pirogov Memorial Medical University, Vinnytsya, Pirogov Street 56, 21018 Vinnytsia, Ukraine; voronkina@vnmu.edu.ua; 4 International Institute of Biomineralogy GmbH, Am St.-Niclas Schacht 13, 09599 Freiberg, Germany; 5 Faculty of Chemistry, Adam Mickiewicz University, Uniwersytetu Poznanskiego 8, 61-614 Poznan, Poland; 6 IMBE, CNRS, IRD, Aix Marseille University, Station Marine d’Endoume, Rue de la Batterie des Lions, 13007 Marseille, France; alexander.ereskovsky@imbe.fr

**Keywords:** biosilica, sponges, actin, spicules, hierarchical biocomposites, biomimetics

## Abstract

Sponges (phylum Porifera) were among the first metazoans on Earth, and represent a unique global source of highly structured and diverse biosilica that has been formed and tested over more than 800 million years of evolution. Poriferans are recognized as a unique archive of siliceous multiscaled skeletal constructs with superficial micro-ornamentation patterned by biopolymers. In the present study, spicules and skeletal frameworks of selected representatives of sponges in such classes as Demospongiae, Homoscleromorpha, and Hexactinellida were desilicified using 10% HF with the aim of isolating axial filaments, which resemble the shape and size of the original structures. These filaments were unambiguously identified in all specimens under study as F-actin, using the highly specific indicators iFluor™ 594-Phalloidin, iFluor™ 488-Phalloidin, and iFluor™ 350-Phalloidin. The identification of this kind of F-actins, termed for the first time as silactins, as specific pattern drivers in skeletal constructs of sponges opens the way to the fundamental understanding of their skeletogenesis. Examples illustrating the biomimetic potential of sophisticated poriferan biosilica patterned by silactins are presented and discussed.

## 1. Introduction

Biosilica is one of the main globally distributed biominerals, present in a broad diversity of microorganisms, protists, diatoms, sponges, and plants (for overview see [1,2,3,4,5]). Sponges (phylum Porifera), with a more than 800 MYR-long history [6,7], represent an outstanding source of biosilica-based skeletal constructs, found in numerous representatives of such classes as Hexactinellida, Demospongiae, and Homoscleromorpha. The sizes of such biosilica formations in sponges range from several micrometers or millimeters up to 3 m in length [8]. At the same time, the sophisticated ornamentation features of their surface are striking in their structural diversity, especially in the case of spicules (microscleres) and reticulate skeletons with a complex geometry and ordered symmetry (for an overview, see [9,10,11,12]). The main functions of biosilicates of a poriferan origin are to create a microporous, mechanically stable skeleton for the optimal distribution of a number of special cells, and an aquiferous system responsible for the flow of water with the accompanying natural feed and oxygen [13]. Some, especially the hook-like microscleres, help retain the organic matrix of the sponge body, while others, with a ray-like morphology (i.e., anchoring spicules), specialize in retaining the entire sponge skeleton in muds and sandy bottoms [14,15,16]. The inorganic chemistry of such forms of poriferan biosilica is not so complex as their structural diversity suggests. Most skeletal constructs of silica-producing sponges are made of pure, amorphous silica with inclusions of Na and K [17]; however, in the case of some deep-sea glass sponges, calcitic nanocrystals have also been found within highly specialized spicular formations (for details, see [18]). In contrast, the organic phases reported within sponges’ glassy skeletons and spicules have been a hotly debated topic since 1888 [19,20] up to the present day [3,9,16,21,22,23]. According to the enzymatic view, such highly specialized biomolecules as silicateins, glassins, hexaxilins, and perisilins (for an overview, see [23]) are responsible for biosilicification as well as spicule formation in sponges. Alternatively, the view that the process is based on corresponding activities of such structural biopolymers as chitin, collagen, and actin is also represented in the literature [3,16,21,22,24,25].

Recently, it was shown with strong evidence (using special phalloidin staining, proteomics, immunostaining, inhibition tests, Western Blotting, Fast Fourier Transformation, HRTEM, and Raman spectroscopy) [24] that axial filaments within diverse skeletal constructs in numerous representatives of two sponge classes (Hexactinellida and Demospongiae) are made of F-actin. It is suggested that the epitaxy of uniquely structured biosilica in sponges is due to the presence, growth, and characteristic branching of actin filaments. Being immured in a glass frame, actin exhibits a patterning function in the sophisticated architecture of poriferan biosilica [22]. Similar results have been obtained previously for diatoms. For example, in large-sized species such as *Coscinodiscus granii* and *Cyclotella cryptica*, actin has been shown to control biosilica patterning in the frustule on the meso- and micro-scale [26,27].

As recently reported [22,24], actin has been identified within skeletal formations of 11 and 4 representatives of Hexactinellida and Demospongiae, respectively. However, despite the fact that actin was discovered in skeletons of these sponge species, the presence of various structural and molecular features of actin cannot be ruled out, causing this structural protein to be associated with a specific species or genus. To assign actins found in the biosilica skeletal structures of sponges to a special group and to avoid confusion with actins from other organisms, it is proposed here to call them silactins. A similar approach was taken previously with cathepsins: those identified as being involved in biosilicification in sponges were renamed silicateins (for overview see [28]).

The aim of this study is to identify silactins in the spicules and skeletal networks of selected representatives of freshwater and marine demosponges and in hexactinellids and homoscleromorphs. Hypotheses on the functioning of actin in poriferan biosilica and explanations for the formation of complex bioarchitectures and symmetries in sponges will be proposed and discussed. Also included is a discussion of the biomimetic potential of this kind of ancient biocomposites with their highly specific structural ornamentation.

## 2. Materials and Methods

### 2.1. Sample Origins

Freshwater sponges:

*Ephydatia muelleri* (Lieberkühn, 1856) (Demospongiae, Spongillida, Spongillidae) specimens were collected in the Moscow Channel (Moscow region, Russia) in July 2019.

*Lubomirskia baikalensis* (Pallas, 1776) and *Baikalospongia bacillifera* (Dybowsky, 1880) (Demospongiae, Spongillida, Lubomirskiidae) were collected during an expedition in June 2010 on the southeastern coast of Lake Baikal near the Bolshie Koty settlement (51°54′25″ N–105°04′14″ E) at a depth of 10–15 m, using SCUBA diving equipment. The apical parts of specimens over 30–40 cm tall were collected and immediately placed in containers with Baikal water and ice for transportation.

*Metania reticulata* (Bowerbank, 1863) (Demospongiae, Spongillida, Metaniidae) samples were collected in the Negro River (Amazon Central Basin region) during the dry season.

*Drulia uruguayensis* (Bonetto and Ezcurra de Drago, 1968) (Demospongiae, Spongillida, Metaniidae) samples were received from the scientific collection of Museu de Ciências Naturais, Fundação Zoobotânica do Rio Grande do Sul, Porto Alegre, RS, Brazil (MCN-POR1152).

*Ochridaspongia rotunda* (Arndt, 1937) (Demospongiae, Spongillida, Malawispongiidae) demosponges were collected from the locality of Velidab in the eastern part of Lake Ohrid (for details see [29]).

Marine sponges:

*Suberites domuncula* (Olivi, 1792) (Demospongiae, Suberitida, Suberitidae) sponges were collected from Roscoff region (Brittany, France) at a depth of 9–12 m using SCUBA diving equipment.

*Axinella damicornis* (Esper, 1794) (Demospongiae, Axinellida, Axinellidae) sponges were collected in July 2020, in the Gulf of Lion, Mediterranean Sea, at a depth of 14–18 m using SCUBA diving equipment.

*Petrosia ficiformis* (Poiret, 1789) (Demospongiae, Petrosiidae) sponges were collected in July 2020, in the Gulf of Lion, Mediterranean Sea, at a depth of 5–8 m using SCUBA diving equipment.

*Polymastia arctica* (Merejkowsky, 1878) (Demospongiae, Polymastiida, Polymastiidae) was collected in the White Sea, Onega Bay, 64°57′0–65°10′8 N, 35°29′4–35°51′6 E, 9–22 m, in summer 1877.

*Sphaerothylus borealis* (Swartschewsky, 1906) (Demospongiae, Polymastiida, Polymastiidae) was collected in the White Sea by Dr. A. Plotkin, Norway.

*Tethya norwegica* (Bowerbank, 1872) (Demospongiae, Tethyida, Tethyidae) was collected in coastal waters of northern Norway by Dr. A. Plotkin.

*Geodia cydonium* (Linnaeus, 1767) (Demospongia, Geodiida, Geodiidae) sponges were collected in Marseille, France, Cave Coral, Maire island, 43°12′37.60″ N 5°20′24.86″ E.

*Erylus granularis* Topsent, 1904 (Demospongia, Geodiida, Geodiidae) Topsent, 1904, UPSZMC 191573 (PC1384), and *Pachymatisma normani* Sollas, 1888 (Demospongia, Geodiida, Geodiidae) Sollas, 1888, UPSZMC 191572 (PC196) were received from the Museum of Evolution, Uppsala, Sweden (UPSZMC)

*Biemna* sp. (Demospongiae, Biemnida, Biemnidae)—dry-preserved freeze-dried sample was received from Western Australian Museum (WAM Z35650).

*Euplectella aspergillum* Owen, 1841 (Hexactinellida, Lyssacinosida, Euplectellidae)—collected in the Philippines, about 150 m depth, purchased from INTIB GmbH, Freiberg, Germany.

*Pheronema nasckaniense* (Tabachnick, 1990) (Hexactinellida, Amphidiscosida, Pheronematidae)—RV Ichthyander 25°46.5′ W, 86°28.5′ S, 395 v depth.

*Plakortis halichondroides* (Wilson, 1902) (Homoscleromorpha, Homosclerophorida, Plakinidae) specimens were collected in Jamaica in March 2005 from a coralligenous reef at depth of 15 m at Pear Tree Bottom using SCUBA equipment.

*Plakina jamaicensis* (Lehnert and van Soest, 1998) (Homoscleromorpha, Homosclerophorida, Plakinidae) specimens were collected in Jamaica from vertical walls of a coralligenous reef at depth of 28 m at Chalet Caribe, west of Montego using SCUBA equipment.

### 2.2. Sample Preparation and Phalloidin Staining

The isolation of axial filaments from the investigated sponge spicules was performed using the “sliding drop technique” [24]. Selected spicules were first treated with 70% HNO_3_ at room temperature for 72 h for removal of possible organic impurities. Then spicules were rinsed in distilled H_2_O up to pH 6.5, dried in air at room temperature, and placed on Nunc™ Permanox™ (Thermo Fisher Scientific, Rochester, NY, USA) plastic microscope slides (27/75 mm) in small drops of water. After water evaporation, one drop of 10% HF acid was added to each sample; the slide was placed in a Plexiglas Petri dish at an angle of about 10° and closed to prevent HF evaporation. Samples were left for 7–10 h to allow the silica to dissolve. The residual demineralized axial filaments of spicules were then rinsed with water and dried in air. 

For larger amounts of demineralized spicules of *E. muelleri* and *S. domuncula*, dialysis through the membrane was used additionally for purification.

For fluorescence staining of demineralized spicules, Cell Navigator™ F-Actin labeling kits (AAT Bioquest, Pleasanton, CA, USA) were used: *Red Fluorescence* iFluor™ 594-Phalloidin (Cat#22664), *Green Fluorescence* iFluor™ 488-Phalloidin (Cat#22661), and *Blue Fluorescence* iFluor™ 350-Phalloidin (Cat#22660). To prepare a working solution, 10 μL of iFluorTM Phalloidin (Component A) was added to 10 mL of Labeling buffer (Component B). To the demineralized spicules fixed on Nunc™ Permanox™ (Thermo Fisher Scientific) plastic microscope slides, iFluor™ Phalloidin working solution was added in a quantity of 100 µL per sample. Samples were stained for 60 min at room temperature in the dark. Afterwards the plates were carefully washed five times with distilled water to remove excess dye, dried, and observed using light and fluorescent microscopy. Unused iFluor™ Phalloidin stock solution was stored at −20 °C and protected from light.

### 2.3. Digital Microscopy

Organic-freed spicules of freshwater and marine sponges before and after demineralization were observed using a Keyence VHX-7000 digital optical microscope with the following zoom lenses: VHX E20 (magnification up to 100×) and VHX E100 (magnification up to 500×) (Keyence, Osaka, Japan).

### 2.4. Scanning Electron Microscopy (SEM)

Morphology of the spicules and asters isolated from *G. cydonium* as well as tylostyles of *S. domuncula* were analyzed using scanning electron microscope (XL 30 ESEM, Philips, Eindhoven, The Netherlands). Prior to scanning, the samples were coated with a gold layer using the Cressington Sputtercoater 108 auto, Crawley (GB) (sputtering time 45 s). 

### 2.5. Fluorescence Microscopy

Fluorescent microscopy images were obtained using a Keyence BZ-9000 digital optical microscope (Keyence, Osaka, Japan) with the zoom lenses CFI Plan Apo 10× and CFI Plan Apo 40× using DAPI channel (Ex/Em = 360/460 nm) for blue-stained samples, GFP channel (Ex/Em = 470/525) for green-stained samples, TxRed channel (Ex/Em = 560/630) for red-stained samples, and the bright field for comparison and/or overlay.

### 2.6. SDS-PAGE

An amount of 300 µg of demineralized and dialyzed spicules of *E. muelleri* was dissolved in 37.5 µL 0.1 M Tris-HCl (pH 7.1) and 12.5 µL NuPAGE^®^ LDS Sample Buffer (Thermo Fisher Scientific, Carlsbad, CA, USA). The sample was vortexed for 3 h, then placed at −20 °C overnight. The samples were heated at 70 °C for 10 min and then centrifuged (5 min, 10,000× *g*). After, 40 μL (for coomassie blue staining) and 10 μL (for silver staining) of the samples were electrophoresed in mPAGE™ 4–20% Bis-Tris Precast Gel (Merck, Germany). ROTI^®^Mark TRICOLOR (Carl Roth, Karlsruhe, Germany) was used as the marker and Actin from rabbit muscle (Sigma-Aldrich, Burlington, MA, USA) as the standard. The gel was run at 200 V and stained with ROTI^®^Blue Colloidal Coomassie Staining (Carl Roth, Karlsruhe, Germany) and ROTI^®^Black P Silver Staining kit for proteins (Carl Roth, Karlsruhe, Germany).

## 3. Results

### 3.1. Actin within Spicules of Freshwater Demosponges

Phalloidin is a bicyclic heptapeptide toxin isolated from the mushroom *Amanita phalloides* which, with high specificity, binds stoichiometrically to F-actin [30]. Already recognized as a “gold standard F-actin marker” [31], it is able to prevent the depolymerization of actin due to filament stabilization [32], even in paraffin-embedded or formaldehyde-fixed samples [33,34]. Also, proteins including actin which were isolated from the biosilica of diatoms’ cell walls [26,35] and spicules of sponges [22,24], after demineralization with HF, have been shown to survive such harsh treatment and can be stained with diverse phalloidins. The preservation of the stability of actins of various origins after treatment with HF has been experimentally proven (for details, see [24]). To confirm the selectivity of phalloidins for actin identification after the HF treatment of poriferan biosilica, such recognized bioanalytical methods as immunostaining, Western blotting techniques, and Raman spectroscopy have been alternatively and successfully used [24]. Thus, the reliability of using phalloidins to identify actin is beyond doubt [36]. Consequently, in this study, we used three different phalloidin markers to confirm the presence of F-actin-based filaments within spicules of selected representatives of both freshwater and marine demosponges, as well as within hierarchically structured skeletal (dictyonal) frameworks of glass sponges.

The aforementioned freshwater sponges belong to eight families of the class Demospongiae (subclass Heteroscleromorpha, order Spongillida,) and include approximately 250 species [37]. For our investigation, we selected seven species from four different families, some of which, like Spongillidae, are cosmopolitan, while others such as Malawispongiidae and Lubomirskiidae are endemic to ancient lakes (Figure S1).

**Figure 1:**
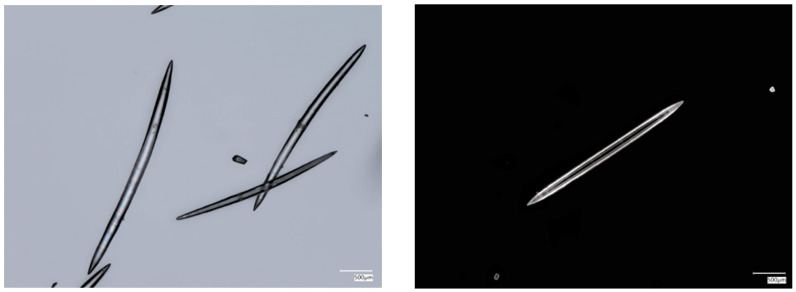
Digital microscopy imagery of *Ephydatia muelleri* freshwater demosponge oxeas with symmetrical tips after removal of organic material using HNO_3_ treatment. Desilicification of such spicules with HF led to isolation of organic axial filaments, which were identified as F-actin (see Figure 2).

**Figure 2:**
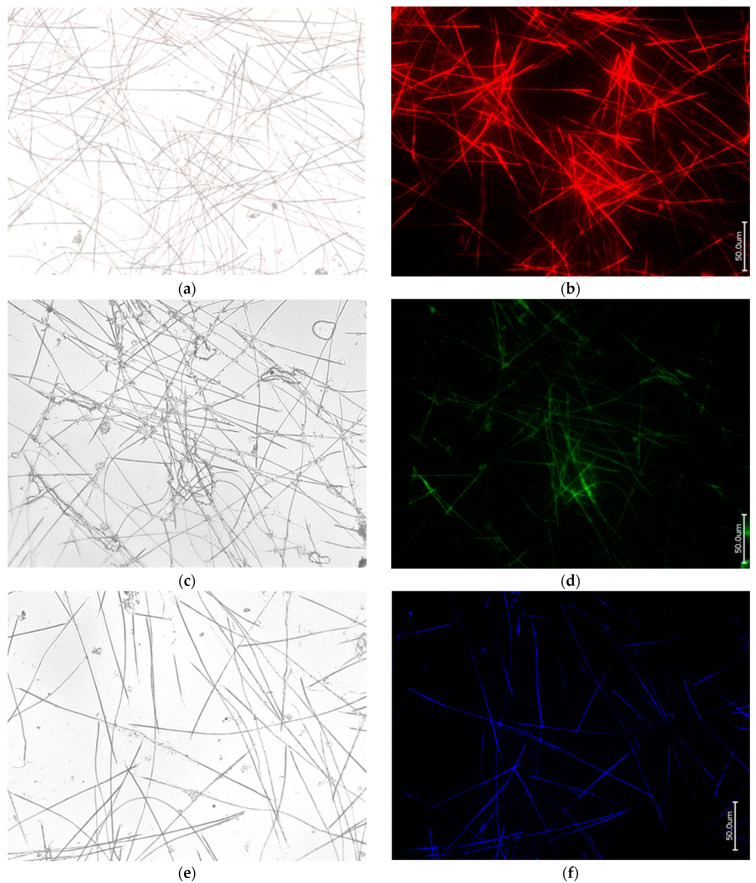
Bright field (**a**,**c**,**e**) and fluorescence microscopy imagery of the axial filaments obtained after demineralization of oxeas of *E. muelleri* freshwater demosponge using HF under the conditions of the “sliding drop technique” [24] and stained with 594-Phalloidin (**b**); also with 488-Phalloidin (**d**) and 350-Phalloidin (**f**) for comparison.

**Figure 3:**
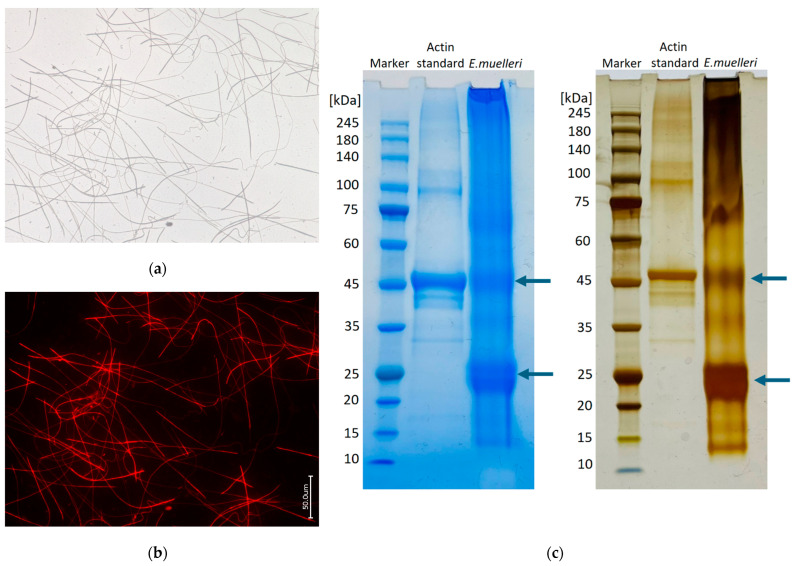
Bright field (**a**) and fluorescence microscopy (**b**) imagery of the axial filaments of *E. muelleri* demosponge oxeas isolated in bulk after HF treatment, dialyzed, and finally stained with 594-Phalloidin. (**c**) SDS-PAGE: arrows indicating the actin (45 kDa) and silicatein (25 kDa) bands well visible after both Coomassie blue (left gel) and silver reagent (right gel) staining of the axial filaments sample *of E. muelleri* under study.

The demineralization of organic-freed spicules, called oxeas, isolated from the worldwide-distributed *Ephydatia muelleri* freshwater demosponge (Figure 1) as a typical representative of the Spongillidae family using both the “sliding drop technique” [22,24] and in bulk treatment with 10% HF, led to the obtaining of corresponding axial filaments (see Figure 2 and Figure 3, respectively). These fibrillar structures were identified as F-actin filaments using characteristic phalloidin staining (Figure 2b,d,f and Figure 3b). Moreover, the presence of actin together with silicateins within these formations was confirmed using SDS-PAGE (Figure 3c). Previously, only silicateins have been recognized as the main proteins localized in axial filaments of demosponges and being responsible for biosilicification (for a modern overview, see [23]). The data obtained are in good accordance with those reported for axial filaments of *Spongilla lacustris*, another broadly distributed representative of freshwater demosponges, where both proteins have also been identified using SDS-PAGE [24].

**Figure 4:**
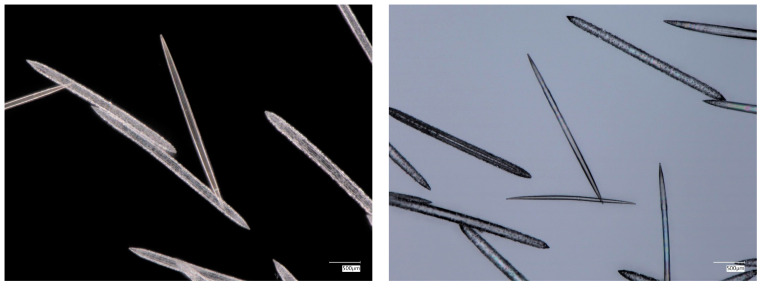
Digital microscopy images of organic-freed acantoxeas and oxeas isolated from the endemic *O. rotunda* freshwater demosponge. Demineralization of such spicules with HF led to isolation of organic axial filaments, which were identified using such diverse phalloidin indicators as F-actin (see Figure 5).

**Figure 5:**
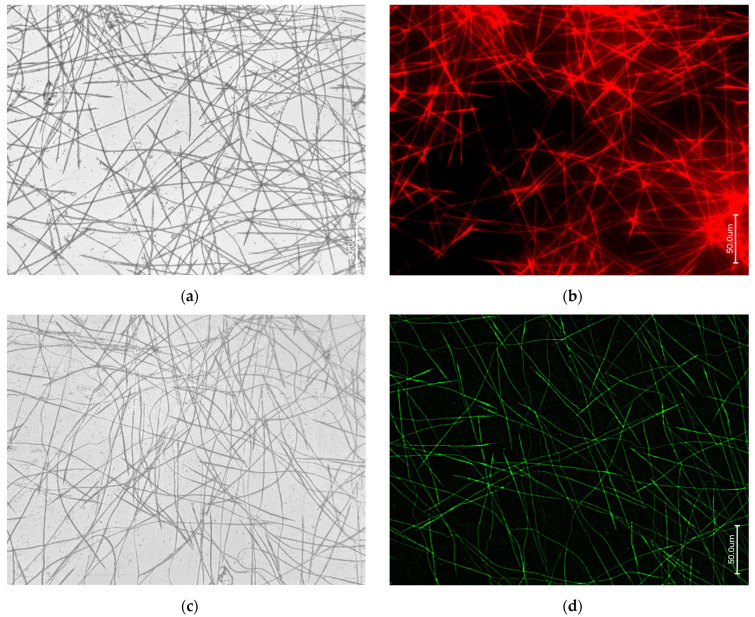

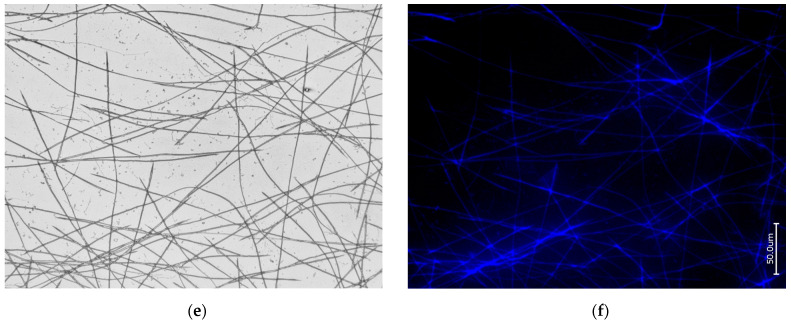
Bright field (**a**,**c**,**e**) and fluorescence microscopy imagery of the axial filaments obtained after demineralization of acantoxeas and oxeas of the *O. rotunda* freshwater demosponge with HF under the conditions of the “sliding drop technique” [24] and stained for comparative purposes with 594-Phalloidin (**b**), 488-Phalloidin (**d**), and 350-Phalloidin (**f**).

For comparative purposes, with the aim of identifying F-actin, we also investigated axial filaments isolated from spicules of such freshwater Amazonian demosponges as *Metania reticulata* and *Drulia uruguayensis* (both of the Metaniidae family), two sponges of the Lubomirskiidae family (*L. baikalensis* and *B. bacilifera*), and the endemic *Ochridaspongia rotunda* (Malawispongiidae) demosponge inhabiting Ohrid lake in North Macedonia and Albania. The results are presented, respectively, in Figure 4, Figure 5 and Figures S2–S9.

Thus, all representatives of the freshwater demosponges considered in this study, which belong to diverse families and inhabit different and distant geographical regions, produce spicules with axial filaments which certainly contain F-actin. This does not exclude the presence of other proteins that are associated with actin or are simply present in the axial channels of spicules in order to perform their special functions, for example, to participate in biosilicification.

### 3.2. Actin within Spicules of Marine Demosponges

Among 30 orders of Heteroscleromorph marine demosponges [38], we selected representatives of seven families from seven orders for our study. The results are presented in Figure 6, Figure 7 and Figure 8 and Figures S10–S14.

**Figure 6:**
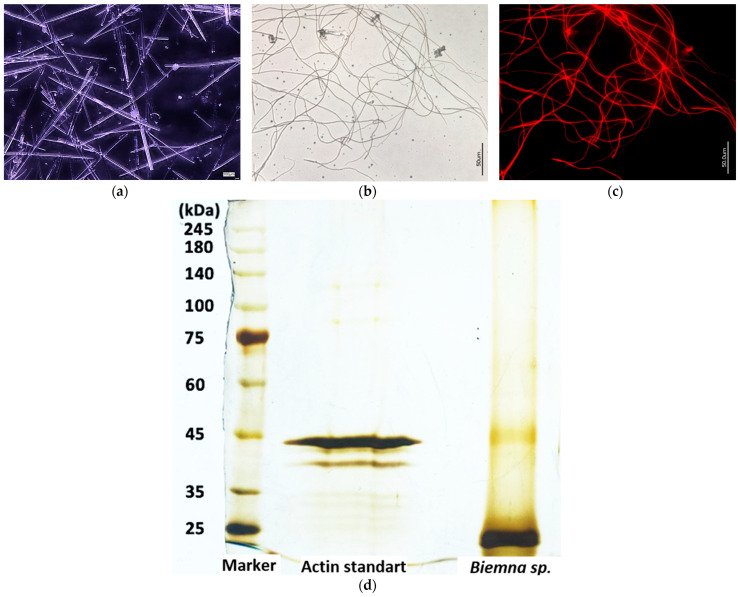
Bright field images of *Biemna* sp. marine demosponge spicules (**a**) and their axial filaments isolated in bulk after HF treatment (**b**). Fluorescence microscopy image (**c**) of dialyzed axial filaments stained with 594-Phalloidin showing the red color characteristic for phalloidin labeled F-actin. (**d**) SDS-PAGE: bands indicating the presence of both actin (45 kDa) and silicateins (25 kDa) in axial filaments extracted after HF-based desilicification of *Biemna* sp. remain well visible after silver reagent staining in two selected samples. For comparison, see Figure 3c.

Thus, axial filaments isolated from spicules of the marine demosponge *Biemna* sp. are also made of both actin and silicateins (Figure 6). The SDS-PAGE data obtained are similar to those presented above for the freshwater sponge *E. muelleri* (see Figure 3c).

**Figure 7:**
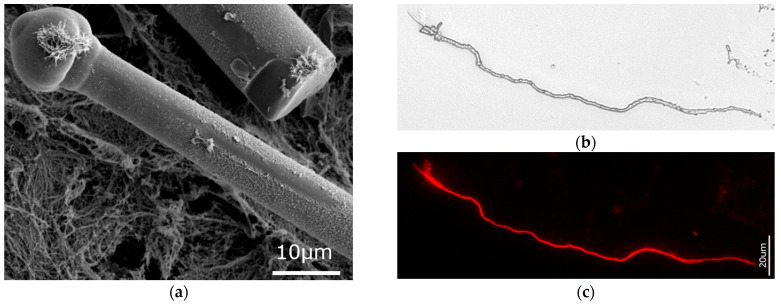
HF-based desilicification of the *S. domuncula* marine demosponge tylostyle (**a**) led to isolation of the axial filaments (**b**), which were identified as F-actin using 594-Phalloidin staining (fluorescence microscopy image (**c**)). F-actin branching of the axial filament fragment within the “club-like” structure is well visible. See also Figure S10.

Another focus of this research was the marine demosponge *Suberites domuncula*, which for many years served as a model organism for the study of silicateins. The discovery of these biosilica-related proteins has been repeatedly reported in the literature (for an overview, see [28,39,40]), but the existence of actin inside the spicules of this sponge as well as the potential involvement of actin was not appreciated or not observed by previous researchers. Figure 7 presents with strong evidence the actin-based nature of the axial filament isolated from this sponge species using the techniques described above. Even the branching—typical for F-actin [41]—of the axial filament fragment originally located within the spicule “club” became visible after corresponding staining with 594-phalloidin. It is suggested here that this kind of branching is responsible for the patterning of the biosilica with respect to the formation of the “club-like” structure.

**Figure 8:**
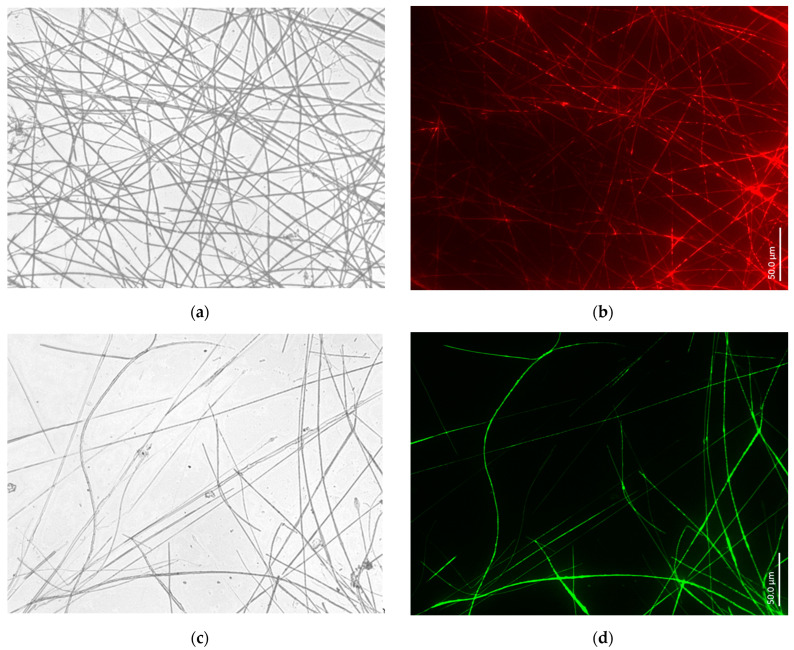

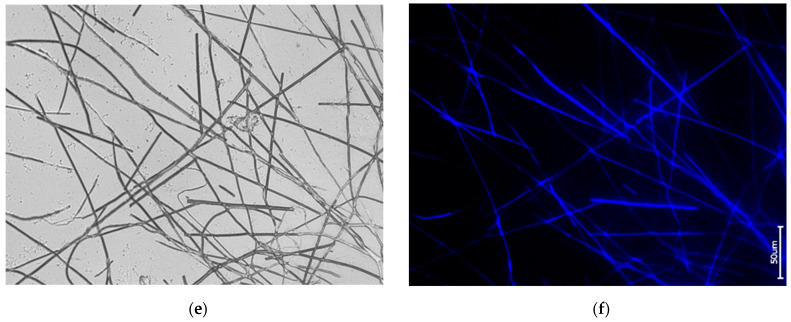
Bright field images of axial filaments isolated from spicules of marine demosponges *P. arctica* (**a**), *S. borealis* (**c**), and *T. norvegica* (**e**) using HF-based treatment as presented above (see Figure 5). Right: fluorescence microscopy images of respective species’ axial filaments stained with 594-Phalloidin (**b**), 488-Phalloidin (**d**), and 350-Phalloidin (**f**).

Similar results (Figure 8) concerning the identification of such axial filaments of spicules as F-actin-based filaments have also been obtained in the case of such strongly psychrophilic Arctic marine demosponges as *Polymastia arctica* (Polymastiidae), *Sphaerothylus borealis* (Polymastiidae), and *Tethya norvegica* (Tethyidae). Analogously to freshwater demosponges, marine species contain F-actin in their spicules, regardless of their geographical habitat and the specific temperature regime of the corresponding marine environment.

**Figure 9:**
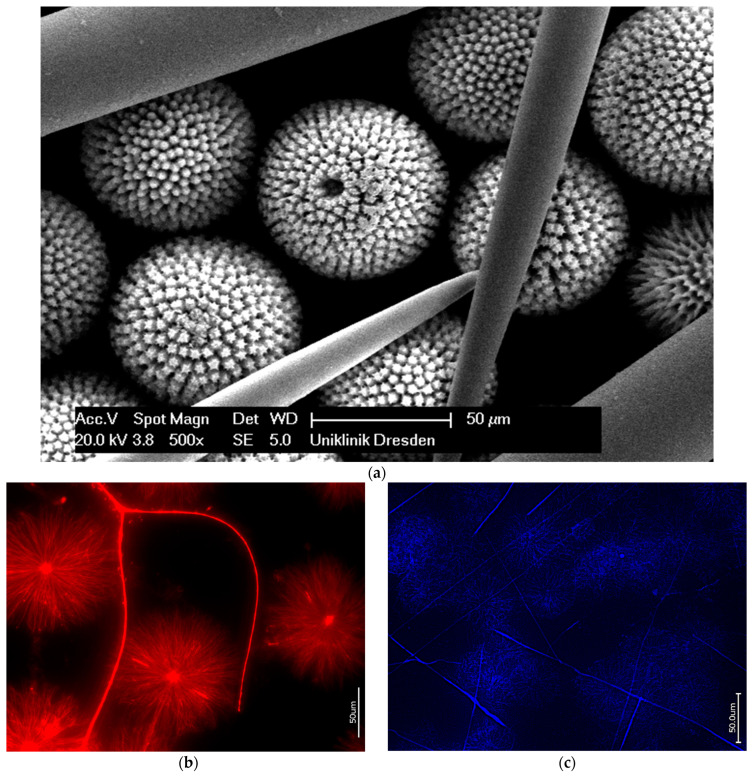
Polybranched microarchitecture of *Geodia cydonium* marine demosponge spicules are well visible, especially in SEM image (**a**). Both types of spicules, radially oriented sterrasters as well as linear megascleres after demineralization using HF, show the presence of correspondingly structured axial filaments, which have been identified as F-actin-based filaments through specific staining with 594-Phalloidin for *Erylus granularis* (Geodiidae) (**b**) and 350-Phalloidin for *G. cydonium* (**c**).

In contrast to the simple structured spicules of the demosponges described above, marine sponges belonging to the family Geodiidae can present even up to four types of mm-sized large spicules known as megascleres in combination with small µm-sized spicules (microscleres) [42]. Traditionally, the ball-shaped sterrasters with sizes of 30–560 µm, with their sophisticated surface micro-ornamentations (Figure 9), are the most striking, and thus researchers have been motivated to investigate the peculiarities of their structural organization. Despite the lack of experimental evidence for the presence of silicateins in these sterrasters, these proteins have previously been proposed as the only organic template [21]. 

However, the results of our study on the desilicification of sterrasters and megascleres from the *Geodia cydonium, Erylus granularis,* and *Pachimatisma normani* demosponges, as typical representatives of the Geodiidae family, demonstrate that the organic phase within them belongs to F-actin (Figure 9b,c). These data echo previously reported findings concerning the presence of actin in *Geodia* biosilica [24]. The fact of the existence of this kind of radially oriented actin filaments is beyond doubt, and they will be discussed in detail below (see Section 4: Discussion).

### 3.3. Actin in the Skeleton of Glass Sponges

The basic triaxonic (six-rayed) symmetry of the skeletal formations found in a diverse range of more than 600 species of glass sponges (Hexactinellida) is one of the characteristic structural features [43,44]. It is well recognized that these sponges produce microporous biosilica-based 3D hierarchical constructs with highly sophisticated network-like geometries [3,15,45]; however, the identity of the biopolymer that may be responsible for the patterning of such structures is still under investigation [23].

**Figure 10:**
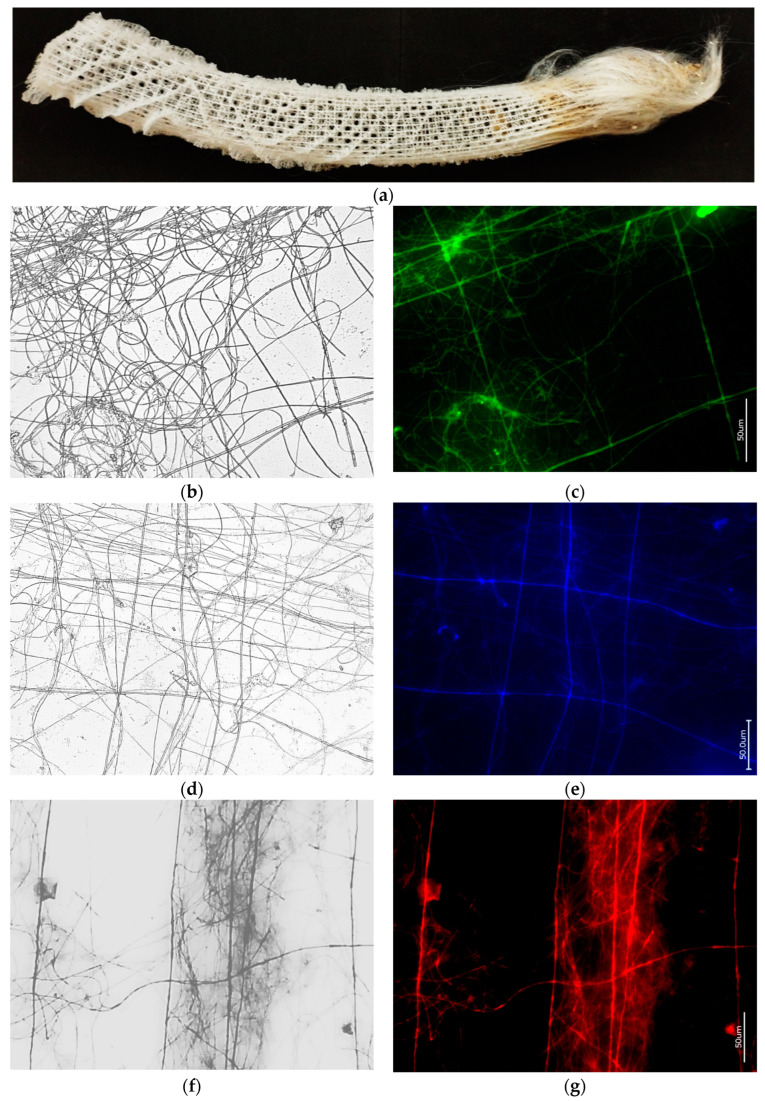
Cell-free 18 cm-long skeleton of *E. aspergillum* glass sponge (**a**) used in the study. Bright field (**b**,**d**,**f**) images of selected skeletal fragments demineralized with HF, with characteristic square geometry of organic filaments. These filaments are identified as F-actin structures using fluorescence microscopy after staining with 488-Phalloidin (**c**), 350-Phalloidin (**e**), and 594-Phalloidin (**g**), corresponding to the bright field images.

Figure 10 presents the results of HF-based desilicification, using the “sliding drop technique” [24], of selected fragments isolated from the *Euplectella aspergillum* glass sponge. For demineralization, fragments of the square architecture were precisely selected and cut out from the glass skeleton with a scalpel. This architecture remained visible after demineralization (see Figure 10b,d,f). The corresponding square-formed elements were clearly stained with diverse phalloidins (Figure 10c,e,g), confirming the presence of F-actin.

**Figure 11:**
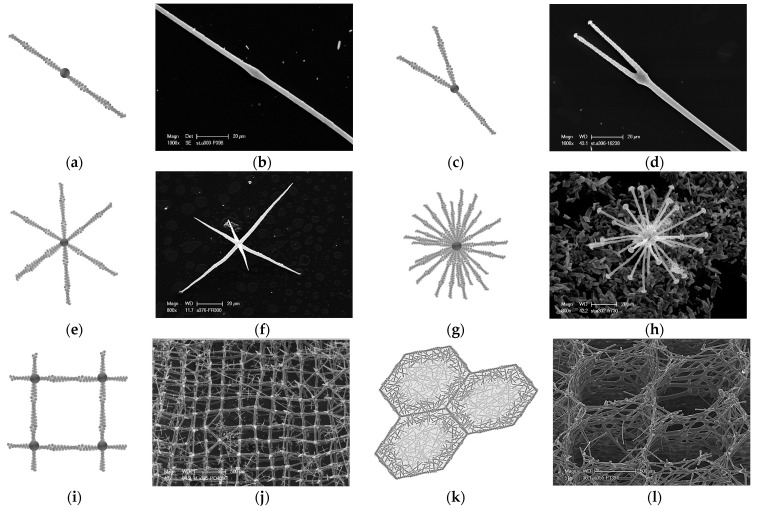
Schematic view of F-actin growth models previously described in the literature vs. siliceous structures observed in Hexactinellida sponges: (**a**) branching of bovine actin [41]; (**b**) uncinate spicule of Tretodictyidae sponge; (**c**) cortical axon branching [47]; (**d**) discoscopule spicule (Hexactinellida, Tretodictyidae); (**e**) actin branching in lamellipodia of *Xenopus laevis* keratocytes [48]; (**f**) oxyhexactin spicule (Hexactinellida, Euretidae); (**g**) astrocytes actin branching in rat nervous system [49]; (**h**) discohexaster spicule (Hexactinellidae, Tretodictyidae); (**i**) endothelial actine cytoskeleton in mouse retina [46]; (**j**) farreoid skeleton (Hexactinellida, Farreidae, *Farrea* sp.); (**k**) honeycomb actin structures in mouse lenses [50] and within diatom frustule [26]; (**l**) honeycomb skeleton of Aphrocallistidae glass sponge *Aphrocallistes* sp. (see also [22]).

At first glance, the discovery of this kind of square architecture of actin filaments in the skeleton of the glass sponge under study seems unexpected. However, actin structures of this type have been described previously, for example, in the endothelial actin cytoskeleton in mouse retinas [46] (Figure 11). The results obtained in our work confirm previously published data on the identification of actin filaments in various hexactinellid species [22,24], but differ from those recently published for the *Euplectella curvistellata* and *Vazella pourtalesii* glass sponges [23]. We do not exclude that the difference in the results obtained is due to different methods for isolating the corresponding proteins. 

As in the SDS-PAGE analysis of the axial filaments of a hexactinellid origin [16,24], in the case of the studied *E. aspergillum* glass sponge, no silicateins were found.

## 4. Discussion

It has recently been shown [22,24,25] that actin as a unique pattern driver leads to the occurrence of superficial ornamentation and specific network connectivity (monaxons, triaxons, and tetraxons) in certain sponge species, which represent more than 46 and 80 morphotypes in Hexactinellida and Demospongiae, respectively. The experimental data presented here strongly confirm that F-actin is the main biosilica patterning biopolymer in a diverse range of simple structured spicules in freshwater and marine demosponges (Figure 1, Figure 2, Figure 3, Figure 4, Figure 5, Figure 6, Figure 7 and Figure 8 and Figures S2–S14) and in hierarchically structured skeletal networks of glass sponges (Figure 10 and Figure S15) and spicules of Homoscleromorphs (Figures S16 and S17). The following are the arguments supporting this conclusion.

(a) Genomic data. There is no evidence of silicatein genes, but those for glassin, as well as collagens and actins, have been reported in the genome of the reef-building psychrophilic glass sponge *Aphrocallistes vastus* (order Sceptrulophora) [51]. Also, in the genome of the Mediterranean *Oopsacas minuta* (order Lyssacinosida) glass sponge, there is no evidence of silicatein, silintaphin, or galectin genes, but actin and glassin genes have been recently reported [52].
(b) Data on inhibition of actin polymerization. It is well recognized that latrunculin B binds to actin monomers and inhibits F-actin polymerization [53]. In recent experiments involving the cultivation of the *Spongilla lacustris* freshwater demosponge from its gemmules, it was shown with strong evidence that actin inhibition by latrunculin B prevents spicule formation [24]. In the samples of hatched gemmules, in the presence of latrunculin B, siliceous spicules never appeared; however, the young sponges grew. To our best knowledge, there are no data on the inhibitory effects of latrunculins against the biosynthesis or self-assembly of silicateins. Consequently, the occurrence of silicateins within sclerocytes of demosponges did not lead to the formation of spicules, but the absence of actin had a decisive impact on spiculogenesis. Put simply, the implication is no actin, no spicules!
(c) Data on structural features characteristic only of actin. Such phenomena known from structural biology as bifurcation, dichotomic growth, and branching represent characteristic features only of actin [48,54]. They are also responsible for the formation of a broad range of higher-order 3D suprafilamentous structures of F-actin: bundles, aggregates, branched, cross-linked, and dendritic filamentous constructs [55,56,57] (see Figure 10). It should be noted that the micrometer size and the quantity of actin filaments that have been isolated from the skeletal formations of diverse demosponges (see Figure 9 as an example) and hexactinellids (see the corresponding images in [22,24]) are not surprising. For example, up to 500 actin filaments have been found in the actin bundles in bristle sprouts of *Drosophila* fruit flies [58]. Also, in the same organism, the bristle cell extension is supported by up to 400 µm-long F-actin bundles assembled together [59,60]. The unique surface ornamentation and sophisticated microarchitecture of some star-like microscleres in demosponges (i.e., sterrasters of *Geodia* sponges) [11,21,24] may seem somewhat extraordinary, and the possible participation of radially oriented actin in this kind of spiculogenesis seems doubtful in principle. However, such a radial structural orientation has already been reported for intracellular actins in *Drosophila* S2 and in *Xenopus* XTC cells [61], as well as in filopodia [62] or lamellipodia of motile cells [63], in flagella [64], and in diverse neurons [65,66,67]. Regarding biosilica-producing organisms, the occurrence of radially oriented actin filaments has been reported within the frustules of such diatoms as *Coscinodiscus granii* and *Cyclotella cryptica* [26,27]. A fundamental remark should be made here: none of the above-mentioned proteins involved in biosilicification in sponges (i.e., silicateins, glassins, etc.) or in diatoms (i.e., silaffins, silacidins, etc.) possess structural features similar to those of actin.

A possible mechanism behind actin-driven pattern formation in poriferan biosilification has already been proposed as follows: “The sponge spicule is initially formed in the silicoblast in the form of a silica-free ‘proteic rodlet’, which is produced in a great vacuoles. This axial rodlet was electron-dense and of fibrillary nature, with spiral fibres 70–100 Å in diameter. The axial filament of F-actin does not mineralize itself but rather provides the base for the mineralization around it. Moreover, while the distal tip of the spicule is open F-actin can elongate, thus driving growth of the spicule. This may continue until the closure of the end of the spicule by mineralization, which stops spicule growth” [24]. If this is the case, the intriguing question of the influence of diverse ecological factors, including contamination with metals, on actin polymerization and spicule formation needs to be addressed. Recently, the number of structural anomalies of spicule-like T-shaped, bulbous enlargements, sharply bent, scissor- and cross-like, and bifurcated formations have been studied in *Eunapius fragilis* freshwater demosponges collected at Markovac (Velika Morava river) in Serbia in relation to water quality [68]. The identification of silactins within such spicule malformations may open the door to a better understanding of the principles of biosilica patterning by these actins under changing environmental conditions.

Our identification of F-actin-based axial filaments within spicules of *Plakortis halichondrioides* and *Plakina jamaicensis* (Figures S16 and S17), as typical representatives of more than 130 species [69] which belong to the class Homoscleromorpha [70], is also important. Despite advances in the molecular systematics and evolutionary biology of homoscleromorphs [71,72], the nature and origin of the organic phase within their mostly tetractinal spicules (calthrops) remain unknown. We are hopeful that our results will motivate homoscleromorph researchers to attempt to confirm the structural role of silactins in this class of biosilica-producing sponges as well.

**Figure 12:**
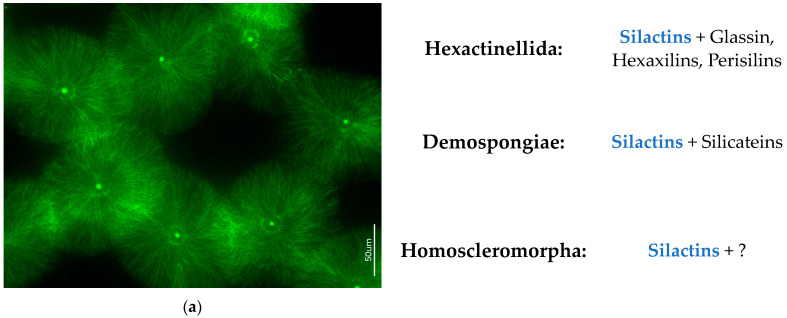
State-of-the-art overview on silactins’ distribution within skeletal structures of three poriferan classes. (**a**) Unique radial orientation of silactin microfilaments of *Pachymatisma normani* (Geodiidae) marine demosponge became well visible after HF-based desilicification of corresponding sterrasters and following staining with 488-phalloidin marker.

An analysis of the literature regarding the role of actin in the biosilicification of various organisms reveals the existence of only six relevant publications. Moreover, only two of them are related to sponges [22,24]; the other four concern unicellular biosilicifying organisms. For example, in these studies, the central role of actin in regulating silica morphogenesis in the diatoms *Rhizosolenia setigera* [73] and *Coscinodiscus granii* [26] as well as in biosilica-producing haptophytes *Prymnesium neolepis* [74] was experimentally confirmed and described. It is hoped that actin will now become a focus of research, and that the sponge-derived silactins will receive particular attention. There are numerous open questions regarding the mechanisms of silactin patterning in diverse poriferan siliceous structures (Figure 11), and there are also plans to carry out in vitro experiments with actin molecules and filaments in the presence of silica sources to create artificial silica-based constructs with and without the addition of silicateins or other recognized substances [23] described previously as biosilicificators (Figure 12). Such studies remain challenging, but crucial for both structural and functional biomimetics. Without a doubt, the modern design strategies of a new generation of engineering materials related to poriferan multiscale hierarchical structures remain a significant trend (Table 1). It is well recognized that they are based on unifying naturally occurring design strategies in sustainable skeletal systems of demosponges, homoscleromorphs, and hexactinellids [45].

## 5. Conclusions

The results of the experimental studies on the detection of actin filaments in biosilica-based formations of sponges representing three poriferan classes clearly show their involvement in spiculogenesis, regardless of the complexity of the glassy bioarchitecture. It was shown that the axial filaments within the spicules of five freshwater and ten marine demosponges contain F-actin as well as silicateins, but only F-actin was identified as a patterning driver of hierarchically structured biosilica in hexactinellids using the example of the *Euplectella aspergullim* glass sponge. For the first time, F-actin has been visualized using highly sensitive phalloidin markers in spicules of Homoscleromorpha, a still poorly investigated class of sponges. To avoid possible confusion and to draw attention to F-actins related to biosilica, it is proposed to call them silactins. The further study of silactins in the skeletal structures of modern as well as fossil sponges—representing the first multicellular organisms on the planet, with a long evolutionary history of more than 800 million years—appears to be an extremely relevant and promising direction of modern bioinspired material science and biomimetics. 

## Figures and Tables

**Table 1:** Biomimetic potential of sophisticated poriferan biosilica.

Biomimetic Directions	References
Mimicking biosintering	[75]
Bioinspired selective laser melting	[76]
Bio-based generative honeycomb design	[22]
Multifunctional design	[77,78]
Bioinspired architecture: next generation of high-performance buildings, skyscrapers, and bridges	[79,80]
Biomimetics of lightweight structural biomaterials	[15,81,82,83,84]
Deep-sea sponge-inspired tubular metamaterials	[85]
Crashworthiness design of a sponge-inspired multicell tube	[86]
Architectured ceramic fibers and damage evolution	[87,88]
Computational modeling of spicule-inspired nested structures	[89,90]
Artificial intelligence-enhanced bioinspiration	[91]
Analytic modeling, finite element modeling, and experimental validation	[92]
3D printing of sponge spicule-inspired flexible bioceramic-based scaffolds	[93,94]
Bioinspired stereolithography	[95]
Bioinspired models for tissue engineering scaffolding	[96]

## Data Availability

Data are contained within the article and Supplementary Materials.

## Supplementary Materials

The following supporting information may be downloaded at https://www.mdpi.com/article/10.3390/biomimetics9070393/s1. Figure S1: Demosponge species selected for this study within the schematic tree of the systematic selection of freshwater sponges. Figure S2: Digital microscopy of *Drulia uruguayensis* (Metaniidae) freshwater demosponge fusiform oxeas after organic material removing using HNO_3_. Figure S3: Bright field (a,c) and fluorescence microscopy images of the axial filaments isolated from 10% HF-demineralized oxeas of *Drulia uruguayensis* (Metaniidae), which have been stained with 594-Phalloidin (b) and with 488-Phalloidin (d). Figure S4: Digital microscopy images of *Metania reticulate* (Metaniidae) freshwater demosponge stronglyoxeas and birotulate gemmoscleres after organic material removing using HNO_3_. Figure S5: Bright field (a,c) and fluorescence microscopy images of the axial filaments isolated from 10% HF-demineralized spicules of *Metania reticulate* (Metaniidae) freshwater demosponge, which was stained with 594-Phalloidin (b) and with 350-Phalloidin (d). Figure S6: Digital microscopy of *Baikalospongia bacilifera* (Lubomirskiidae) freshwater demosponge monaxon megascleres (tilotes) with spiny tips after organic material removing using HNO_3_. Figure S7: Fluorescence microscopy imagery of the axial filaments isolated from 10%HF-demineralized spicules of *Baikalospongia bacilifera* (Lubomirskiidae) freshwater demosponge: (b) 594-Phalloidin stained; (d) 488-Phalloidin stained (f) 350-Phalloidin stained; (a,c,e)—bright field images for comparison. Figure S8: Digital microscopy images of *Lubomirskia baikalensis* (Lubomirskiidae) freshwater demosponge spiny megascleres (acantoxeas) after organic material removing using HNO_3_. Figure S9: Fluorescence microscopy imagery of the axial filaments isolated from with 10% HF-demineralized spicules of *Lubomirskia baikalensis* (Lubomirskiidae) freshwater demosponge: (b) 594-Phalloidin stained; (d) 488-Phalloidin stained (f) 350-Phalloidin stained; (a,c,e)—bright field images for comparison. Figure S10: Fluorescence microscopy imagery of the axial filaments isolated from with 10% HF-demineralized spicules of *Suberites domuncula* (Suberitidae) marine demosponge: (b) 594-Phalloidin stained; (d) 488-Phalloidin stained; (a,c)—bright field images for comparison. Figure S11: Fluorescence microscopy image (b) of the axial filaments isolated from with 10%HF-demineralized spicules of *Axinella damicornis* (Axinellidae) marine demosponge after staining with 594-Phalloidin. (a)—bright field image for comparison. Figure S12: Fluorescence microscopy image (b) of the axial filaments isolated from with 10%HF-demineralized spicules of *Petrosia ficiformis* (Petrosiidae) marine demosponge after staining with 488-Phalloidin. (a)—bright field image for comparison. Figure S13: Fluorescence microscopy image (b) of the axial filaments isolated from with 10%HF-demineralized spicules of *Sphaerotylus borealis* (Polymastiidae) marine demosponge after staining with 594-Phalloidin. (a)—bright field image for comparison. Figure S14: Fluorescence microscopy imagery of the axial filaments isolated from with 10%HF-demineralized spicules of *Tethya norvegica* (Tethyidae) marine demosponge after staining with 594-Phalloidin (b) and 488-Phalloidin (d). (a)—bright field images for comparison. Figure S15: Fluorescence microscopy images of the axial filaments isolated from with 10%HF-demineralized spicules of *Pheronema* glass sponges (a) and stained with 488-Phalloidin (b) and 594-Phalloidin (c). Figure S16: Fluorescence microscopy imagery of the axial filaments isolated with 10%HF-demineralized spicules of Homoscleromorph sponge *Plakina jamaiscensis* (Plakinidae) stained with (b) 594-Phalloidin and (d) with 350-Phalloidin. (a)—bright field image of the axial filaments for comparison. Figure S17: Fluorescence microscopy image (b) of the axial filament isolated from with 10%HF-partially demineralized spicules of Homoscleromorph sponge *Plakortis halichondroides* stained with 594-Phalloidin. (a) Image in bright field for comparison.
