# Supplementary material for: Silactins and Structural Diversity of Biosilica in Sponges
Source: Biomimetics (Basel). 2024 Jun 27;9(7):393. doi: 10.3390/biomimetics9070393 (PMC11274843; doi:10.3390/biomimetics9070393)
Supplement: Supplementary file 1 [file biomimetics-09-00393-s001.zip › biomimetics-3057986-supplementary.pdf]

biomimetics

Article

Silactins and Structural Diversity of Biosilica in Sponges

Hermann Ehrlich [1,2,*], Alona Voronkina [3], Konstantin Tabachnick [4], Anita Kubiak [1,5], Alexander Ereskovsky [6] and Teofil Jesionowski [2,*]

1 Center of Advanced Technology, Adam Mickiewicz University, Uniwersytetu Poznańskiego 10, 61-614 Poznan, Poland; anita.kubiak@amu.edu.pl
2 Faculty of Chemical Technology, Institute of Chemical Technology and Engineering, Poznan University of Technology, Berdychowo 4, 60-965 Poznan, Poland
3 Pharmacy Department, National Pirogov Memorial Medical University, Vinnytsya, Pirogov Street 56, 21018 Vinnytsia, Ukraine; voronkina@vnmu.edu.ua
4 International Institute of Biomineralogy GmbH, Am St.-Niclas Schacht 13, 09599 Freiberg, Germany; tutunnik1999@gmail.com
5 Faculty of Chemistry, Adam Mickiewicz University, Uniwersytetu Poznanskiego 8, 61-614 Poznan, Poland
6 IMBE, CNRS, IRD, Aix Marseille University, Station Marine d'Endoume, Rue de la Batterie des Lions, 13007 Marseille, France; alexander.ereskovsky@imbe.fr
* Correspondence: herehr@amu.edu.pl (H.E.); teofil.jesionowski@put.poznan.pl (T.J.)

Citation: Ehrlich, H.; Voronkina, A.; Tabachnick, K.; Kubiak, A.; Ereskovsky, A.; Jesionowski, T. Silactins and Structural Diversity of Biosilica in Sponges. *Biomimetics* **2024**, *9*, x. https://doi.org/10.3390/biomimetics9070393

Academic Editors: Stanislav N. Gorb, Giuseppe Carbone, Peter Fratzl and Thomas Speck

Received: 29 May 2024
Revised: 23 June 2024
Accepted: 24 June 2024
Published: 27 June 2024

Copyright: © 2024 by the authors. Submitted for possible open access publication under the terms and conditions of the Creative Commons Attribution (CC BY) license (https://creativecommons.org/licenses/by/4.0/).

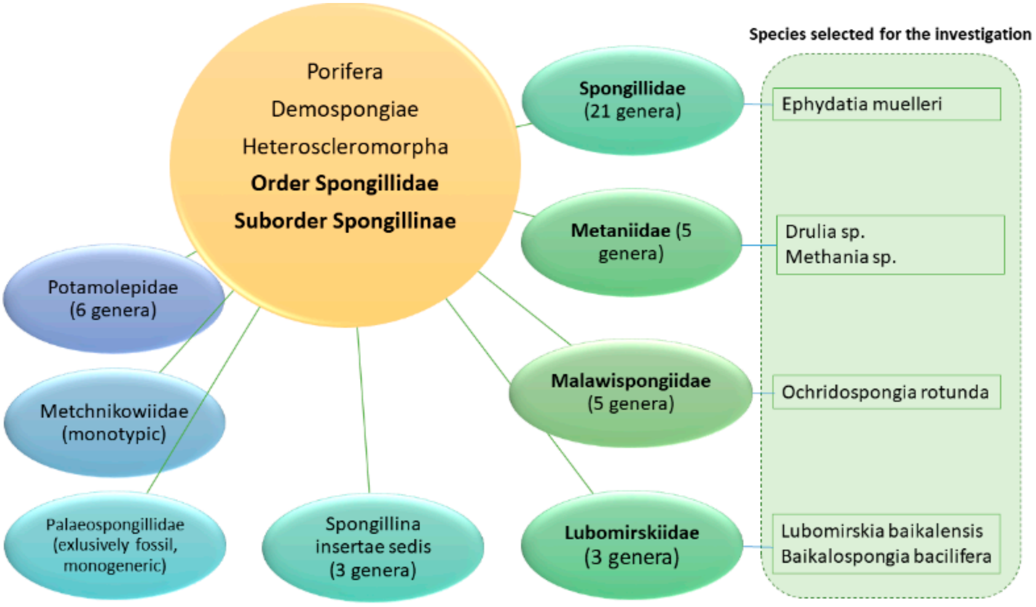

Figure S1. Demosponges species selected for this study within the schematic tree of the systematic of freshwater sponges.

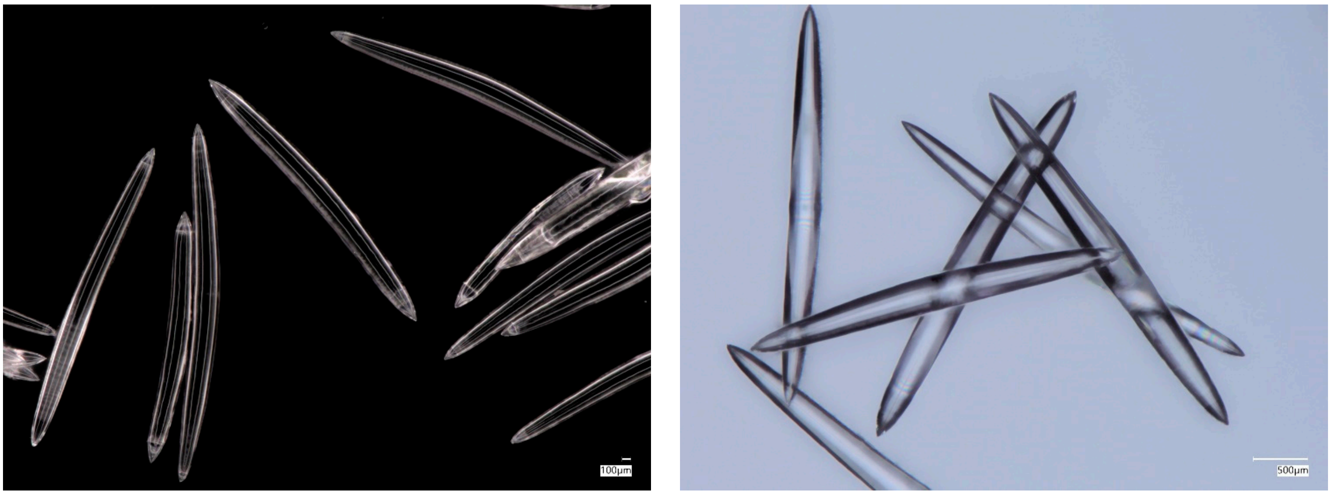

Figure S2. Digital microscopy of *Drulia uruguayensis* (Metaniidae) freshwater demosponge fusiform oxeas after organic material removing using HNO_3.

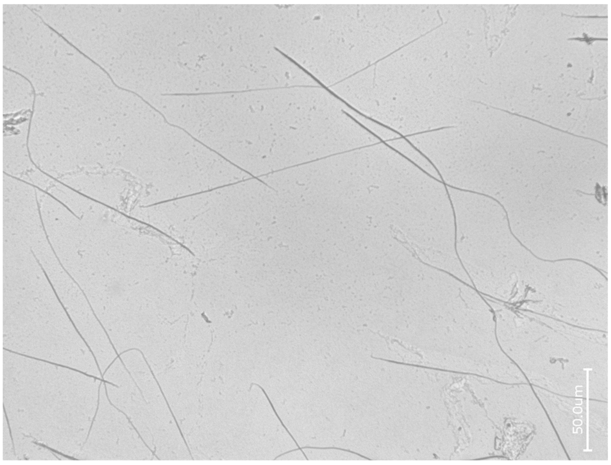

(**a**)

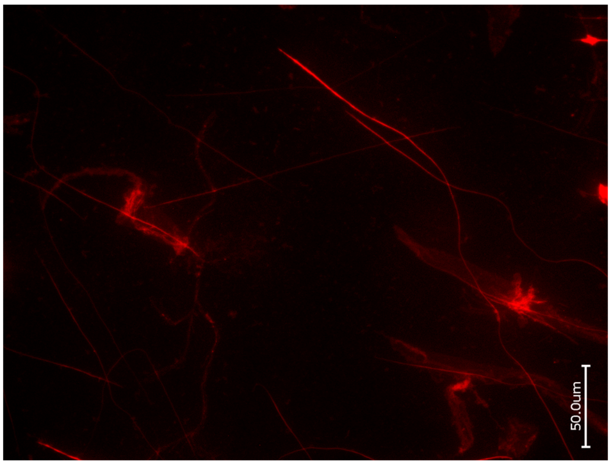

(**b**)

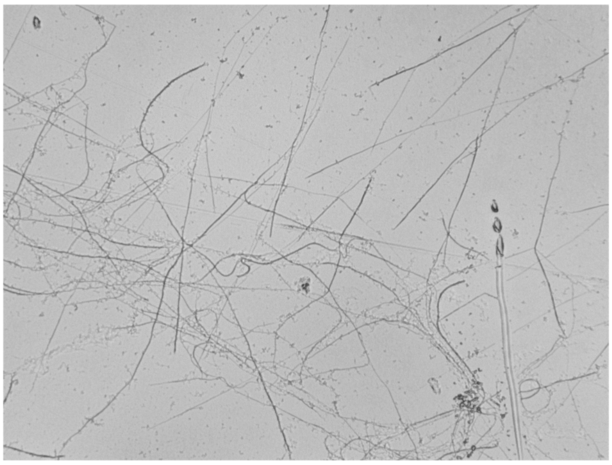

(**c**)

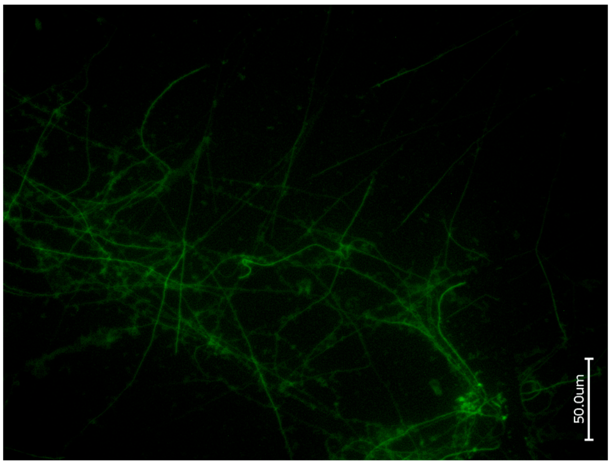

(**d**)

Figure S3. Bright field (**a**) and (**c**) and fluorescence microscopy images of the axial filaments isolated from demineralised using 10% HF oxeas of *Drulia uruguayensis* (Metaniidae), which have been stained with 594-Phalloidin (**b**); and with 488-Phalloidin (**d**).

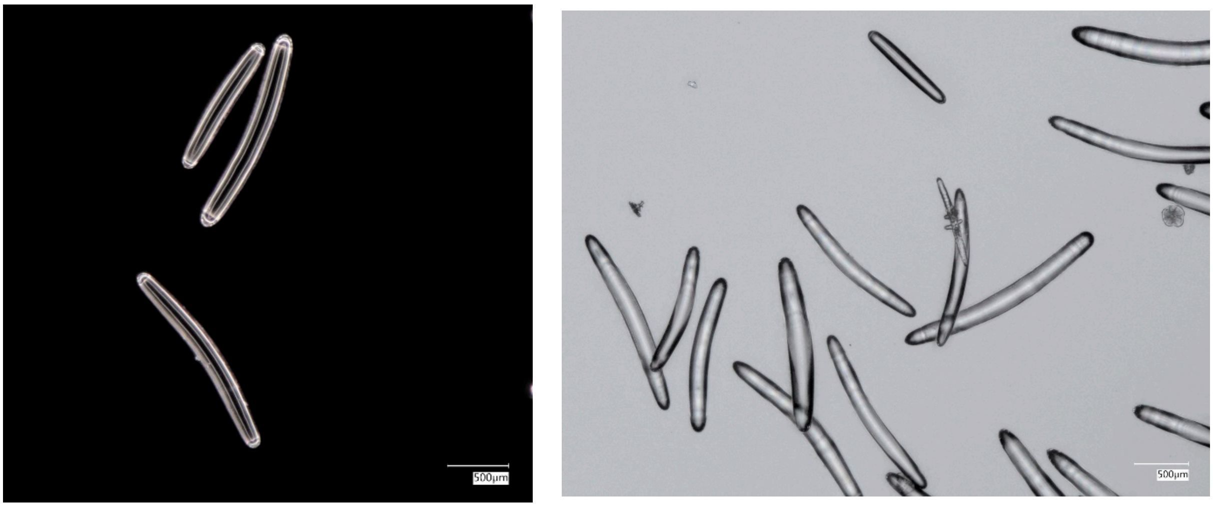

Figure S4. Digital microscopy images of *Metania reticulate* (Metaniidae) freshwater demosponge stronglyoxeas and birotulate gemmoscleres after organic material removing using HNO_3.

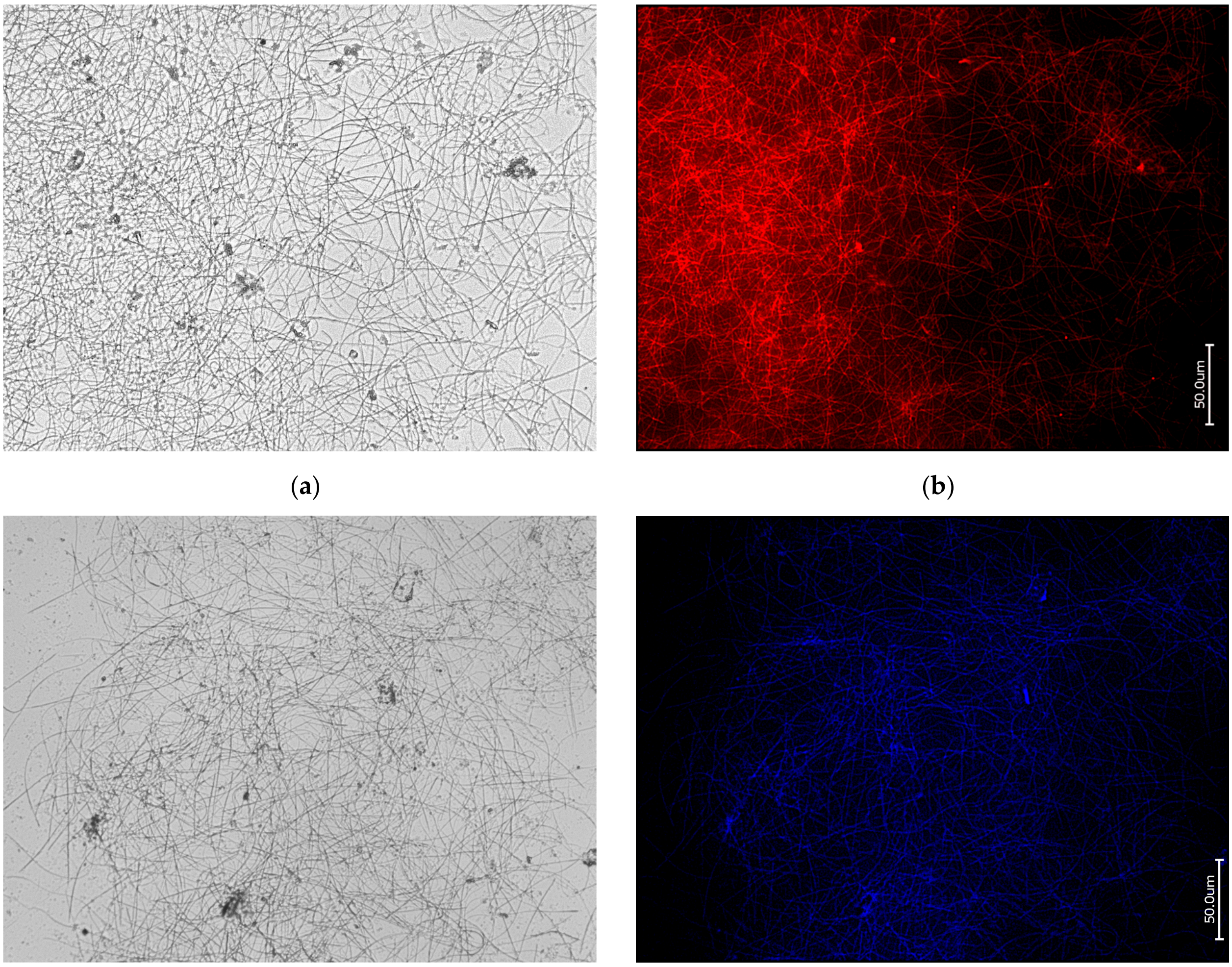

(**a**) (**b**)

(**c**) (**d**)

Figure S5. Bright field (**a** and **c**) and fluorescence microscopy images of the axial filaments isolated from demineralized using 10% HF spicules of *Metania reticulate* (Metaniidae) freshwater demosponge, which have been stained with 594-Phalloidin (**b**); and with 350-Phalloidin (**d**).

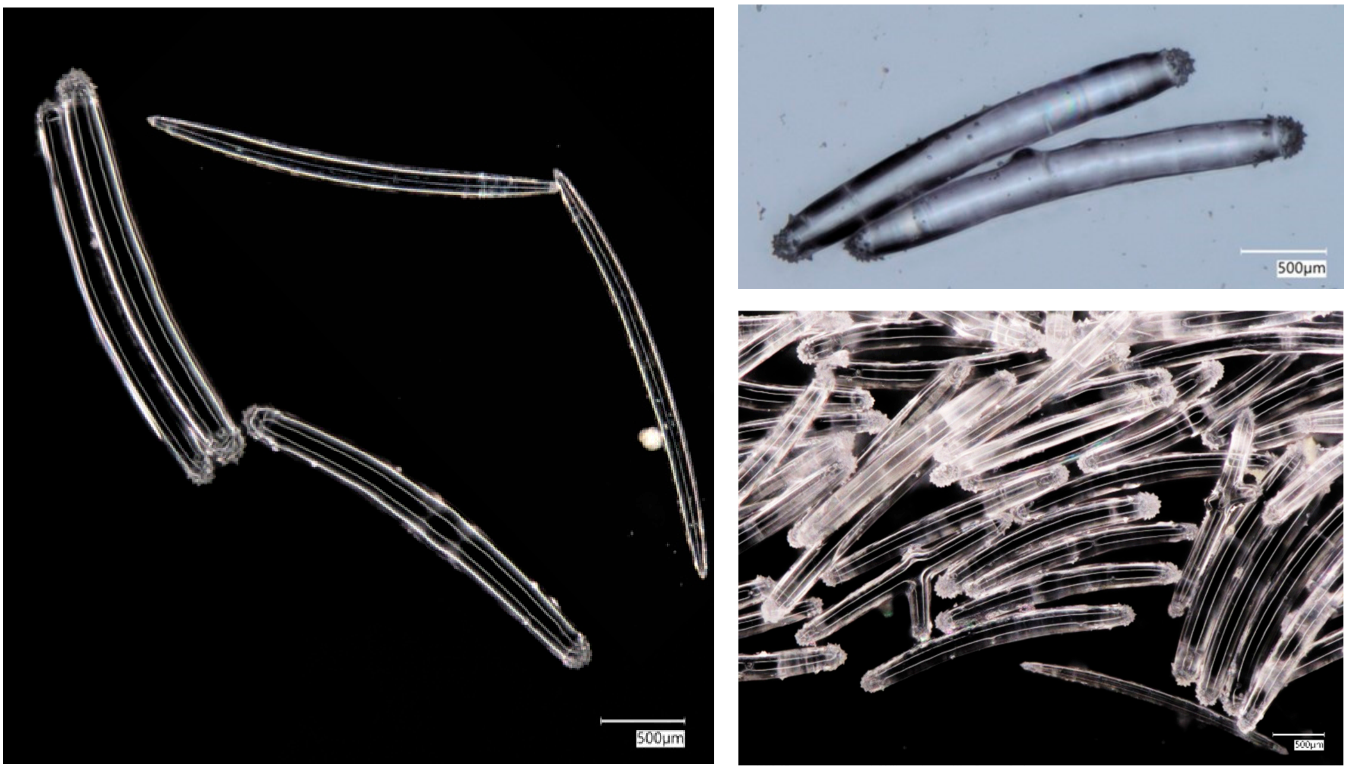

Figure S6. Digital microscopy of *Baikalospongia bacilifera* (Lubomirskiidae) freshwater demosponge monaxon megascleres (tilotes) with spiny tips after organic material removing using HNO_3.

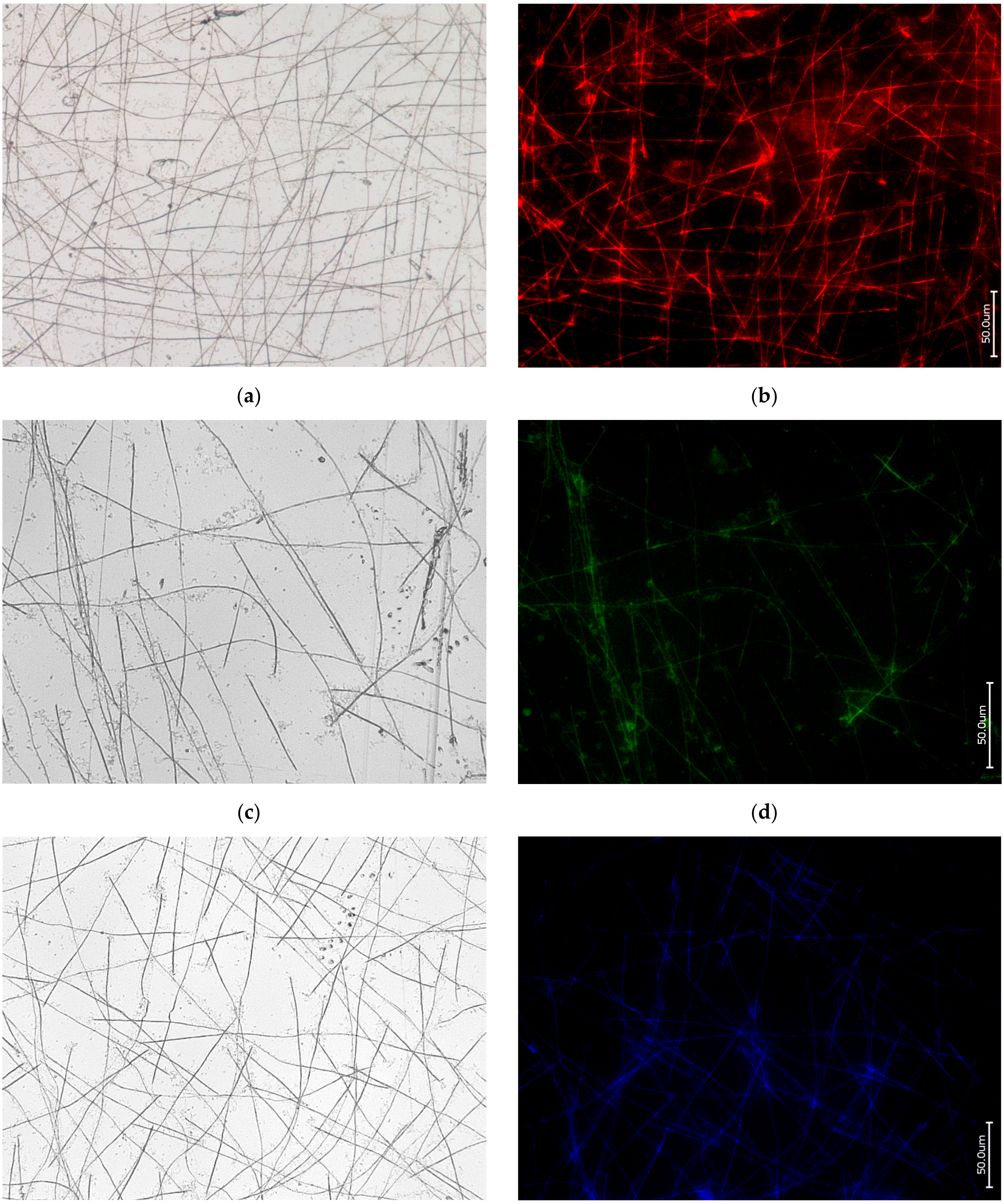

(**a**) (**b**)

(**c**) (**d**)

(**e**) (**f**)

Figure S7. Fluorescence microscopy imagery of the axial filaments isolated from demineralised using 10%HF spicules of *Baikalospongia bacilifera* (Lubomirskiidae) freshwater demosponge: (**b**) 594-Phalloidin stained; (**d**) 488-Phalloidin stained (**f**) 350-Phalloidin stained; (**a**), (**c**), (**e**) – bright field images for comparison.

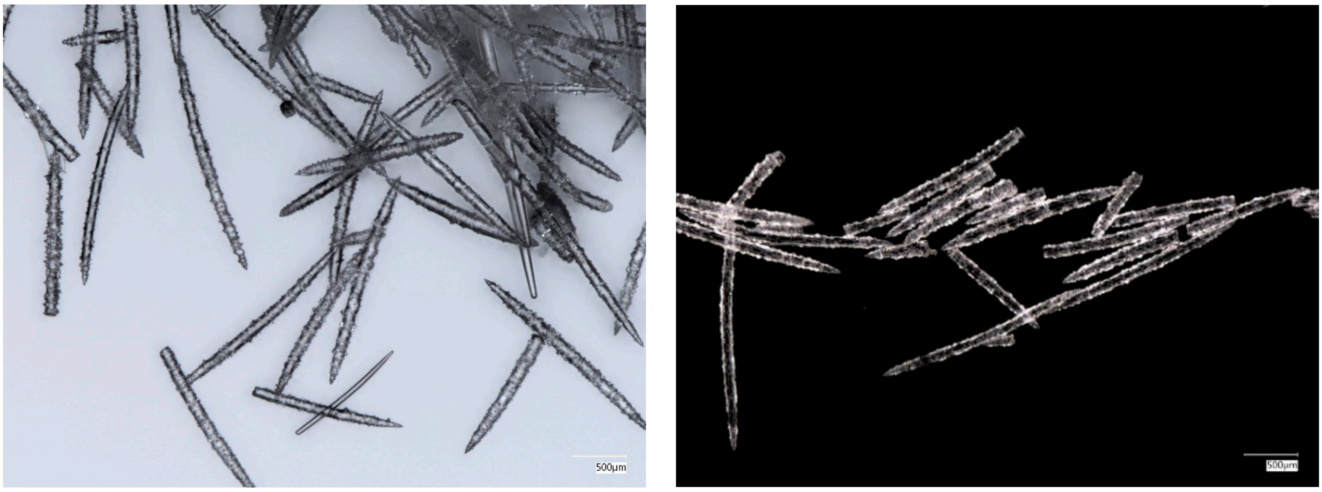

Figure S8. Digital microscopy images of *Lubomirskia baikalensis* (Lubomirskiidae) freshwater demosponge spiny megascleres (acantoxeas) after organic material removing using HNO_3.

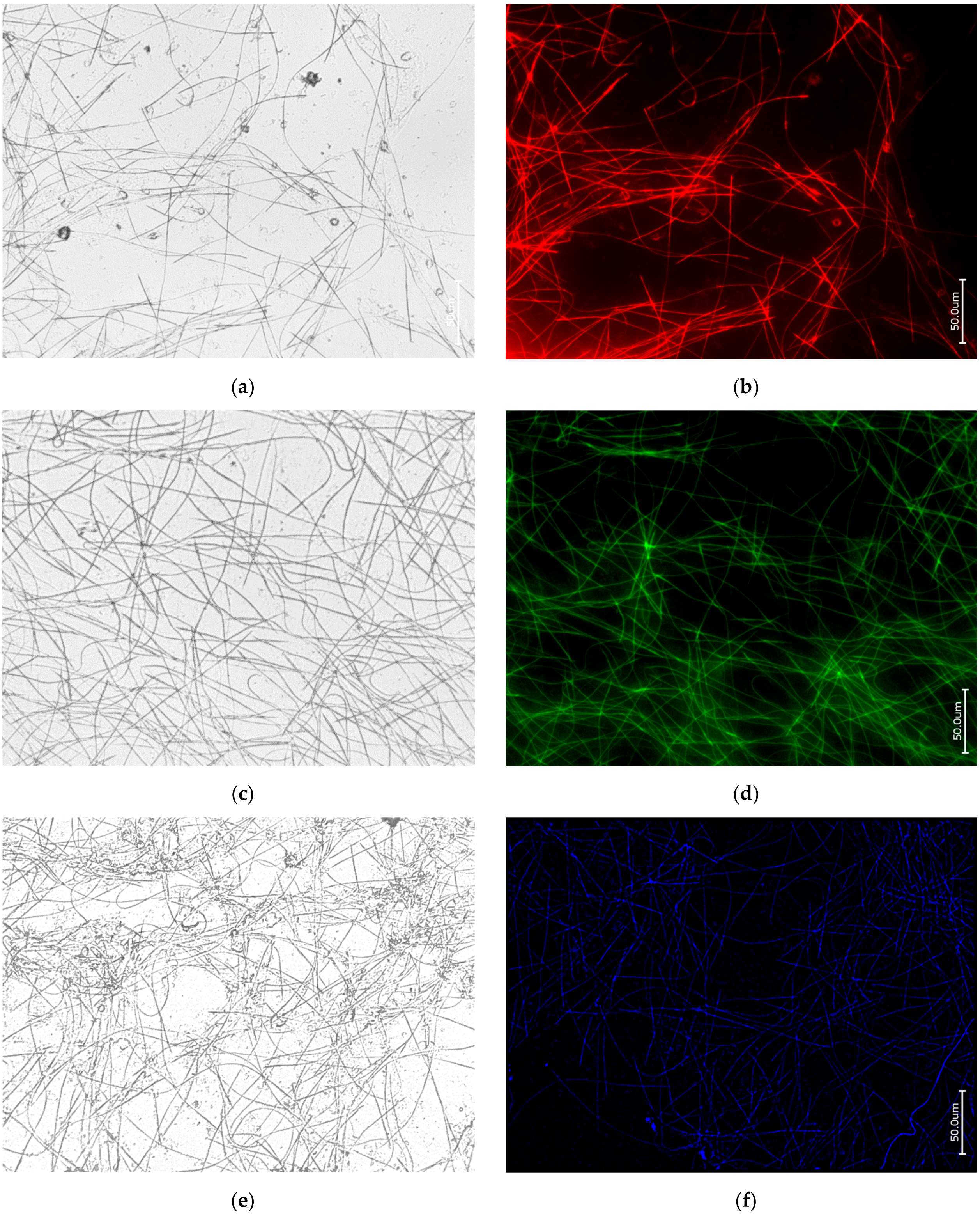

Figure S9. Fluorescence microscopy imagery of the axial filaments isolated from with 10% HF-demineralized spicules of *Lubomirskia baikalensis* (Lubomirskiidae) freshwater demosponge: (**b**) 594-Phalloidin stained; (**d**) 488-Phalloidin stained (**f**) 350-Phalloidin stained; (**a**), (**c**), (**e**) – bright field images for comparison.

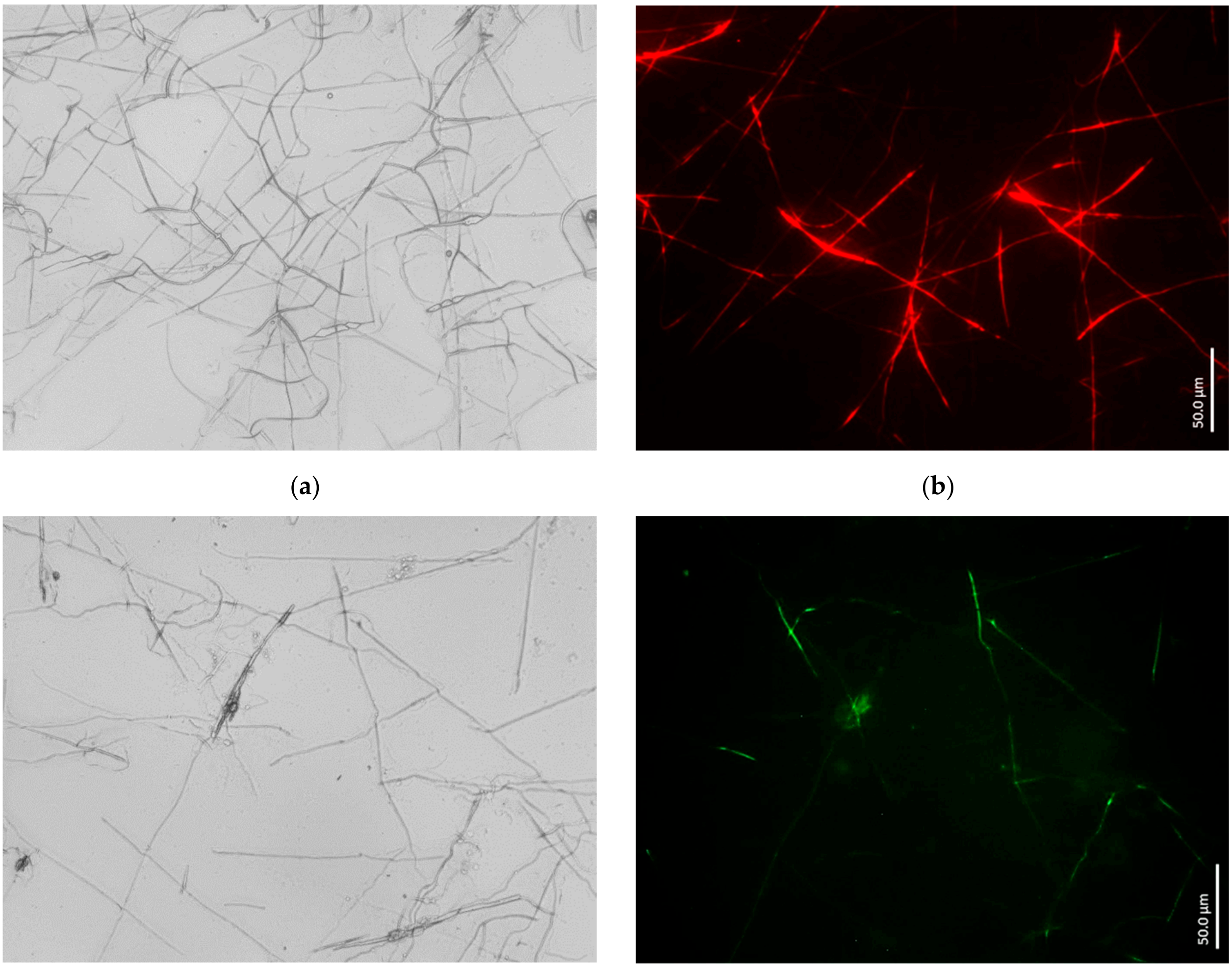

(**a**) (**b**)

(**c**) (**d**)

Figure S10. Fluorescence microscopy imagery of the axial filamrnts isolated from with 10% HF-demineralized spicules of *Suberites domuncula* (Suberitidae) marine demosponge*:* (**b**) 594-Phalloidin stained; (**d**) 488-Phalloidin stained; (**a**), (**c**) – bright field images for comparison.

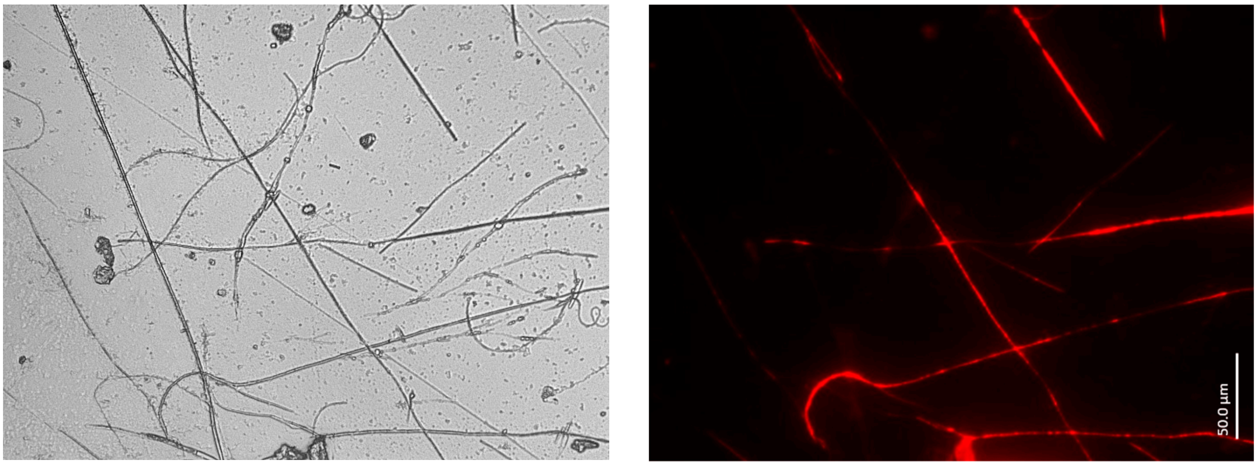

(**a**) (**b**)

Figure S11. Fluorescence microscopy image (**b**) of the axial filaments isolated from with 10%HF-demineralized spicules of *Axinella damicornis* (Axinellidae) marine demosponge after staining with 594-Phalloidin. (**a**) – bright field image for comparison.

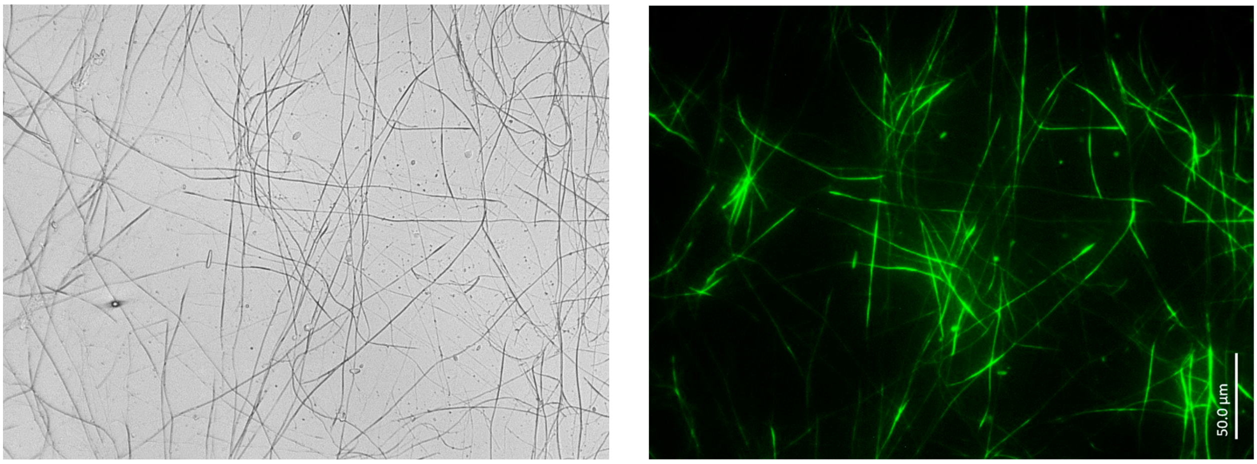

(**a**) (**b**)

Figure S12. Fluorescence microscopy image (**b**) of the axial filaments isolated from with 10%HF-demineralized spicules of *Petrosia ficiformis* (Petrosiidae) marine demosponge after staining with 488-Phalloidin. (**a**) – bright field image for comparison.

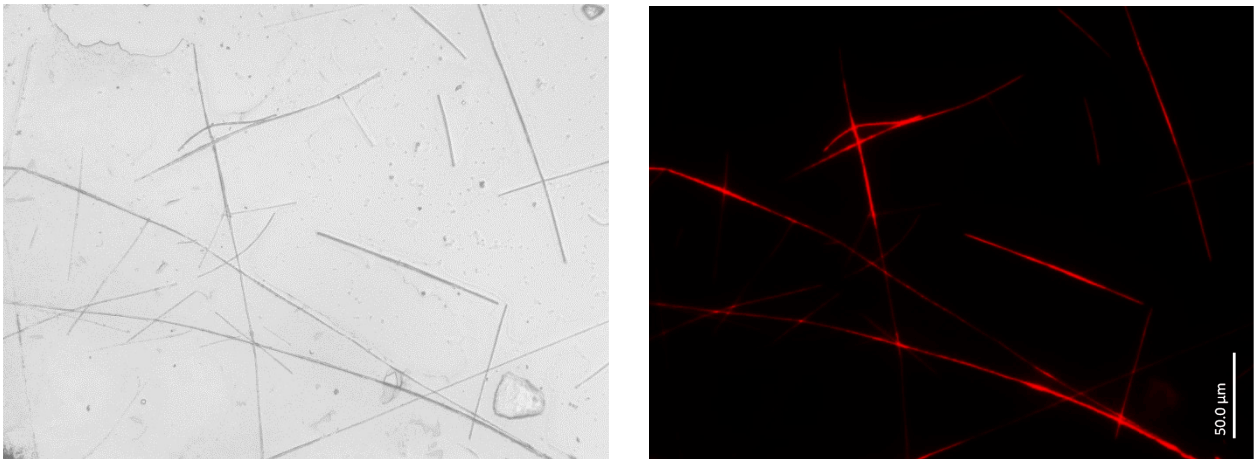

(**a**) (**b**)

Figure S13. Fluorescence microscopy image (b) of the axial filaments isolated from with 10%HF-demineralized spicules of *Sphaerotylus borealis* (Polymastiidae) marine demosponge after staining with 594-Phalloidin. (a) – bright field image for comparison.

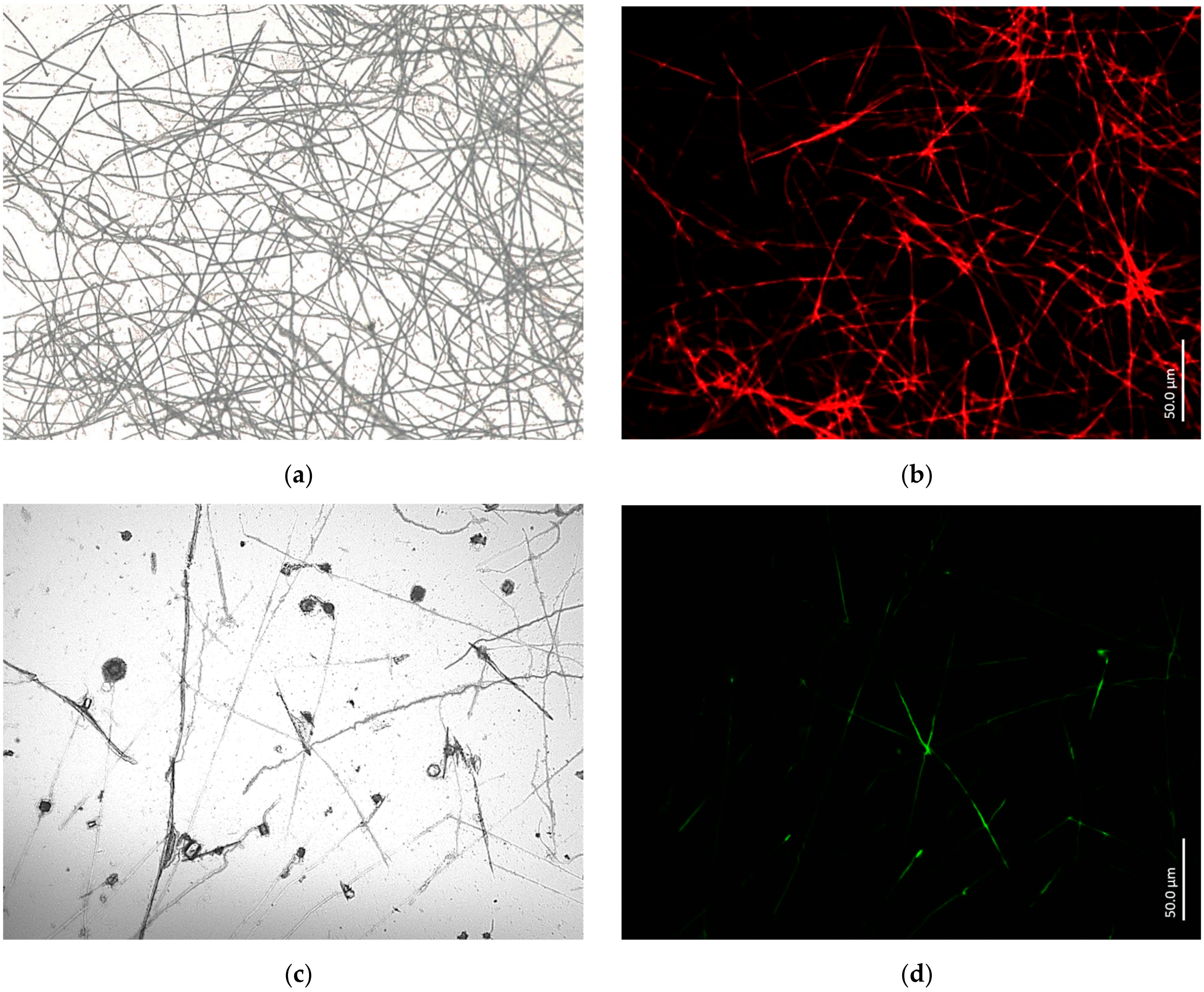

Figure S14. Fluorescence microscopy imagery of the axial filaments isolated from with 10%HF-demineralized spicules of *Tethya norvegica* (Tethyidae)marine demosponge after staining with 594-Phalloidin (**b**) and 488-Phalloidin (**d**). (**a**) – bright field images for comparison.

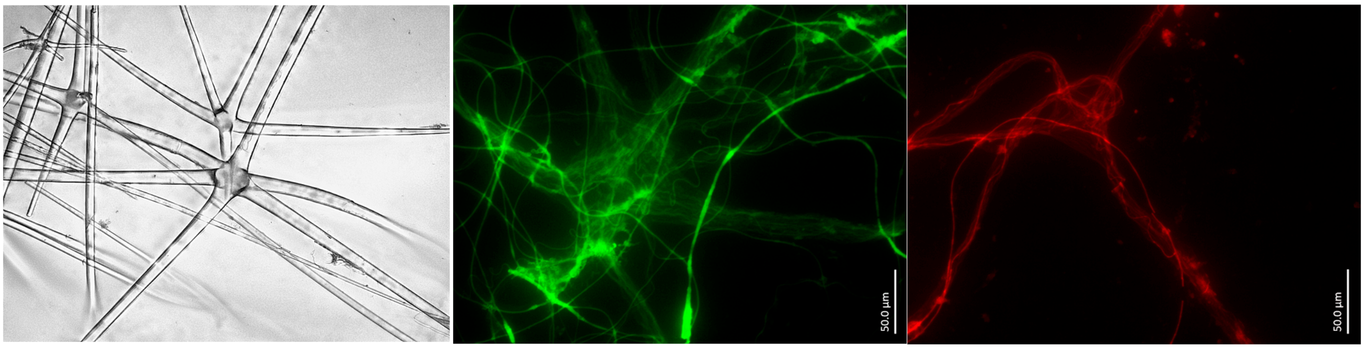

(**a**) (**b**) (**c**)

Figure S15. Fluorescence microscopy images of the axial filaments isolated from with 10%HF-demineralized spicules of *Pheronema* glass sponges (**a**) and stained with 488-Phalloidin (**b**) and 594-Phalloidin (**c**).

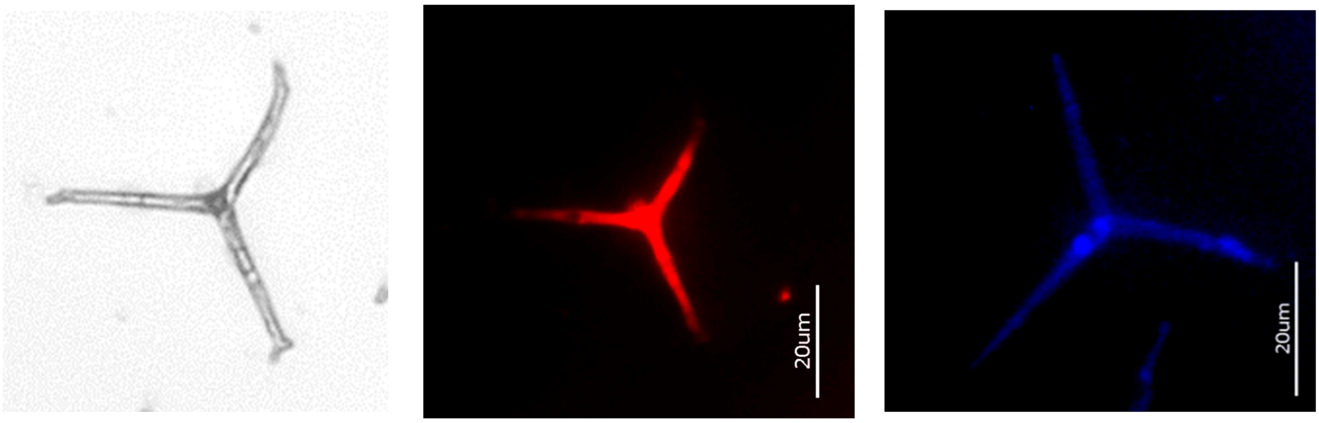

(**a**) (**b**) (c)

Figure S16. Fluorescence microscopy imagery of the axial filaments isolated from with 10%HF-demineralized spicules of Homoscleromorph sponge *Plakina jamaiscensis* (Plakinidae) stained with (**b**) 594-Phalloidin and (**d**) with 350-Phalloidin. (a) —bright field image of the axial filaments for comparison.

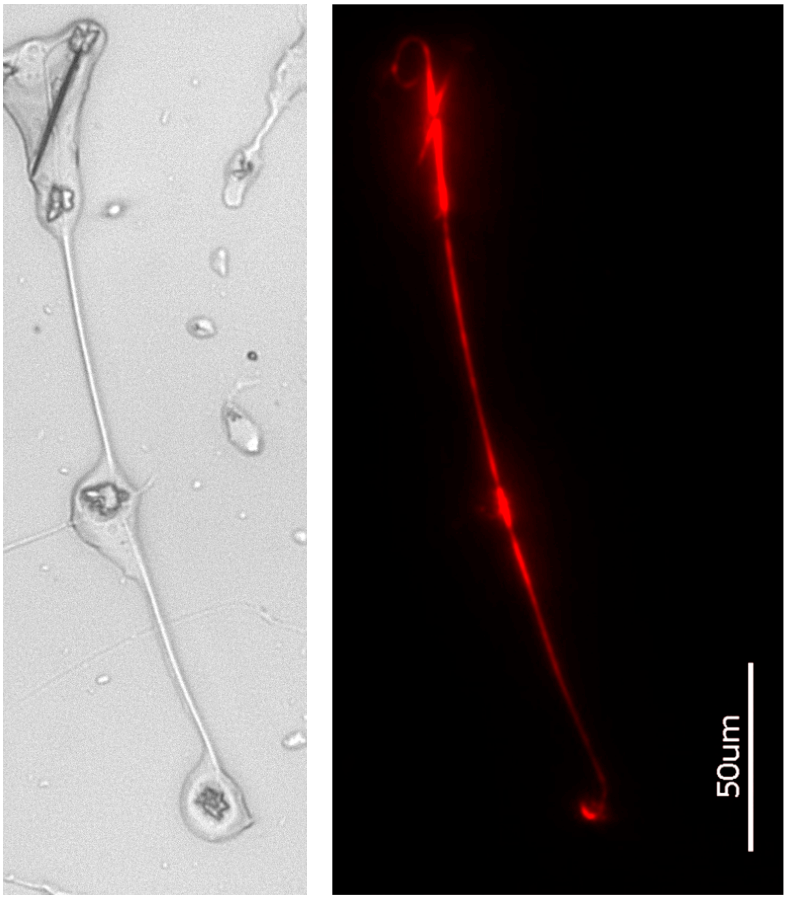

(**a**) (**b**)

Figure S17. Fluorescence microscopy image (**b**) of the axial filament isolated from with 10%HF partially demineralized spicules of Homoscleromorph sponge *Plakortis halichondroides* stained with 594-Phalloidin. (**a**) Image in bright field for comparison.
